# Author Correction: Synaptotagmin-7 outperforms synaptotagmin-1 to promote the formation of large, stable fusion pores via robust membrane penetration

**DOI:** 10.1038/s41467-025-60021-y

**Published:** 2025-05-20

**Authors:** Kevin C. Courtney, Taraknath Mandal, Nikunj Mehta, Lanxi Wu, Yueqi Li, Debasis Das, Qiang Cui, Edwin R. Chapman

**Affiliations:** 1 Howard Hughes Medical Institute and the Department of Neuroscience, University of Wisconsin, 1111 Highland Avenue, Madison, WI 53705 USA; 2 Department of Biochemistry and Molecular Medicine, West Virginia University, Morgantown, WV 26506 USA; 3 Department of Chemistry, Boston University, Boston, MA 02215 USA; 4 Department of Physics, Indian Institute of Technology – Kanpur, Kanpur, 208016 India; 5 Center for Bioanalytical Chemistry, University of Science and Technology of China, Hefei, 230026 China; 6 Department of Biological Sciences, Tata Institute of Fundamental Research, Homi Bhabha Road, Navy Nagar, Colaba, Mumbai, 400005 India

**Keywords:** Synaptic vesicle exocytosis, Lipids, Exocytosis

Correction to: *Nature Communications* 10.1038/s41467-023-42497-8, published online 27 November 2023

In the version of the article initially published, a sample mix-up led to unexpected observations of the syt7 C2AB_2A_ mutation apparently slowing the kinetics of disassembly. Further experimentation has resolved the question and corrected the error.

In the corrected article, the Fig. 4g syt7 C2AB_2A_ blot has been replaced, and the trace for syt7 C2AB_2A_ in Fig. 4i has been updated (for comparison, original and revised panels are available as Fig. 1, below). The source data, full-length gel for Fig. 4g and raw data for graphs in Fig. 4i have been also updated.

In the third paragraph of the “Hydrophobic residues are critical for C2-domain•membrane interaction” section, the text now reading “These results demonstrated that both C2A domains of syt7 contribute comparably to the salt insensitivity (Fig. 4g, i, left panel). … Specifically, the 5 W mutation slowed the disassembly of Ca2 + •syt1 from liposomes after mixing with excess EGTA (Fig. 4h, right panel), and the syt7 4 A, syt7 C2A_2A_B and syt7 C2AB_2A_ mutations significantly increased the disassembly rate (Fig. 4i, right panel), further supporting the importance of loop hydrophobicity” has replaced the original text “These results demonstrated that the C2A domain of syt7 is responsible for the salt insensitivity, whereas the syt7 C2AB_2A_ mutation had no effect (Fig. 4g, i, left panel). We also found, via stopped-flow rapid mixing experiments, that the membrane dissociation kinetics of each of these constructs shared the same trends as the co-sedimentation studies (Fig. 4h, i, right panels). Specifically, the 5 W mutation slowed the disassembly of Ca2 + •syt1 from liposomes after mixing with excess EGTA (Fig. 4h, right panel), and both the syt7 4 A and syt7 C2A_2A_B mutations significantly increased the disassembly rate (Fig. 4i, right panel), further supporting the importance of loop hydrophobicity. Curiously, we found that the syt7 C2AB_2A_ mutation reproducibly slowed the kinetics of disassembly (Fig. 4i, right panel). At present, a mechanism for this perplexing C2AB_2A_ mutation effect is unknown.” The changes are made in the HTML and PDF versions of the article.

Fig. 1 Original and revised Fig. 4g, i

Original Figure:
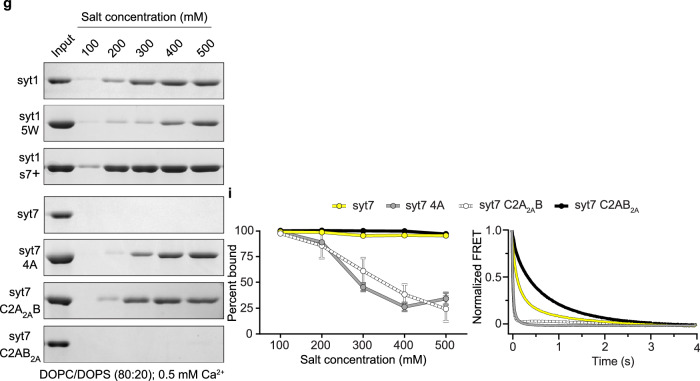


Revised Figure:
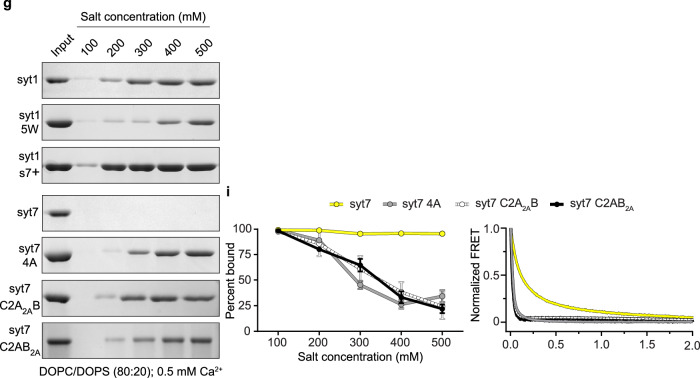


## Supplementary information

Revised Source Data

