# Supplementary material for: Synaptotagmin-7 outperforms synaptotagmin-1 to promote the formation of large, stable fusion pores via robust membrane penetration
Source: Nat Commun. 2023 Nov 27;14:7761. doi: 10.1038/s41467-023-42497-8 (PMC10681989; doi:10.1038/s41467-023-42497-8)
Supplement: Supplementary file 1 — Supplementary Information [file 41467_2023_42497_MOESM1_ESM.pdf]

Supplementary Figures

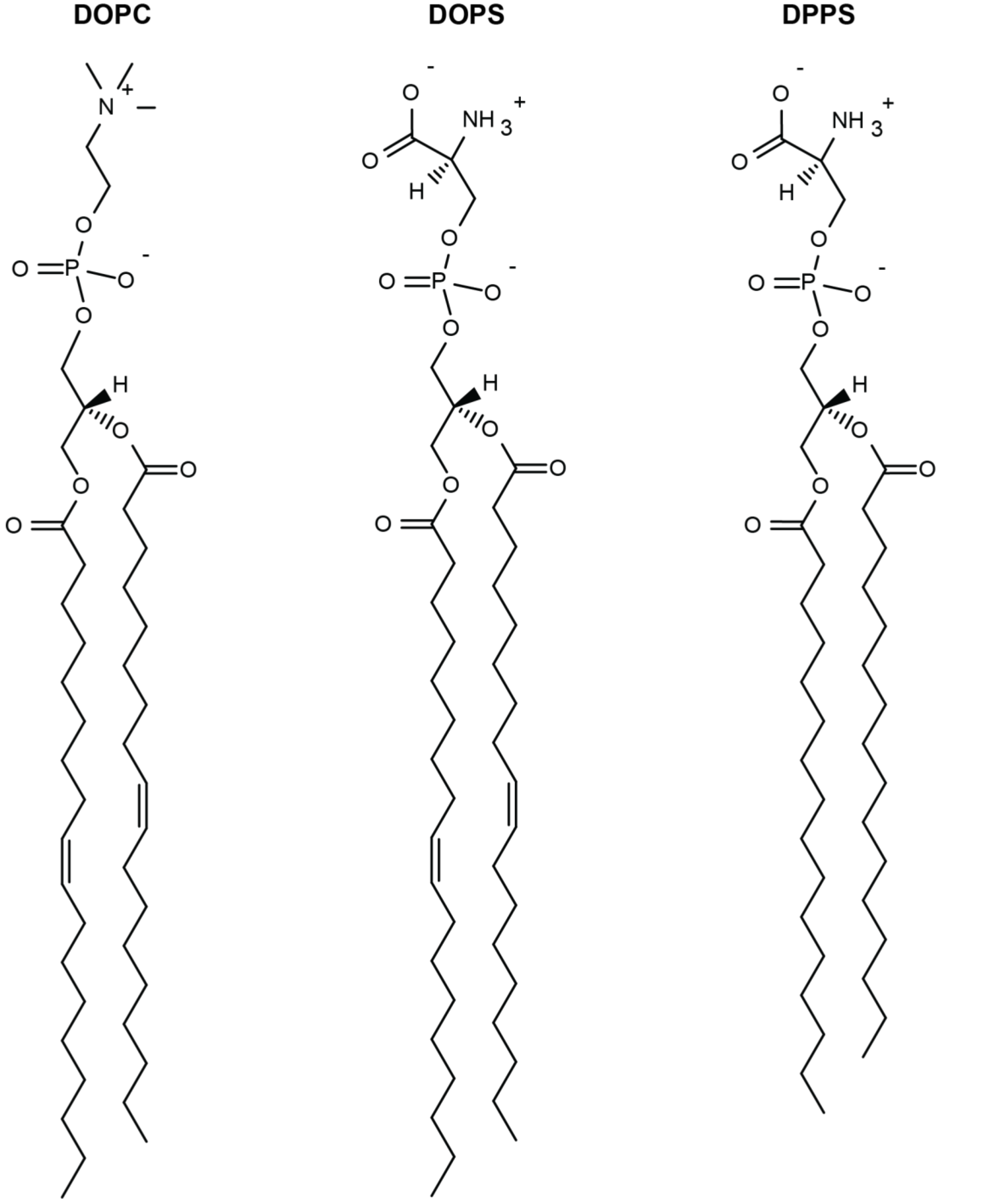

Supplementary Fig. 1. **Phospholipid structures.** Chemical structures of the phospholipids used in this study

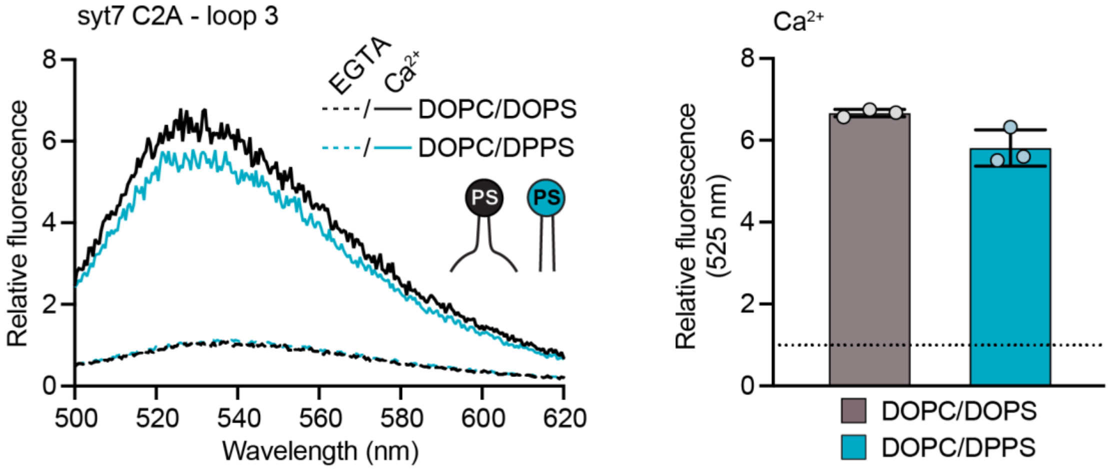

Supplementary Fig. 2. **The C2A domain of syt7 C2AB equally penetrates bilayers containing DOPS and DPPS.** Representative fluorescence emission spectra (*left panel*) of NBD labelled syt7, in the presence (solid lines) and absence (dotted lines) of Ca^{2+}, and liposomes composed of 80:20 DOPC/DOPS (black) or DOPC/DPPS (blue). The syt7 C2AB domain is labeled on loop 3 of the C2A domain at position 229. Quantification of NBD-syt7 C2AB fluorescence emission at 525 nm in the presence of Ca^{2+} and liposomes composed of DOPC/DOPS (black) or DOPC/DPPS (blue), *right panel*. The data are normalized to the EGTA condition, shown as a horizontal black dotted line. Each condition was repeated three times on different days using fresh materials. Error bars represent standard error of the mean.

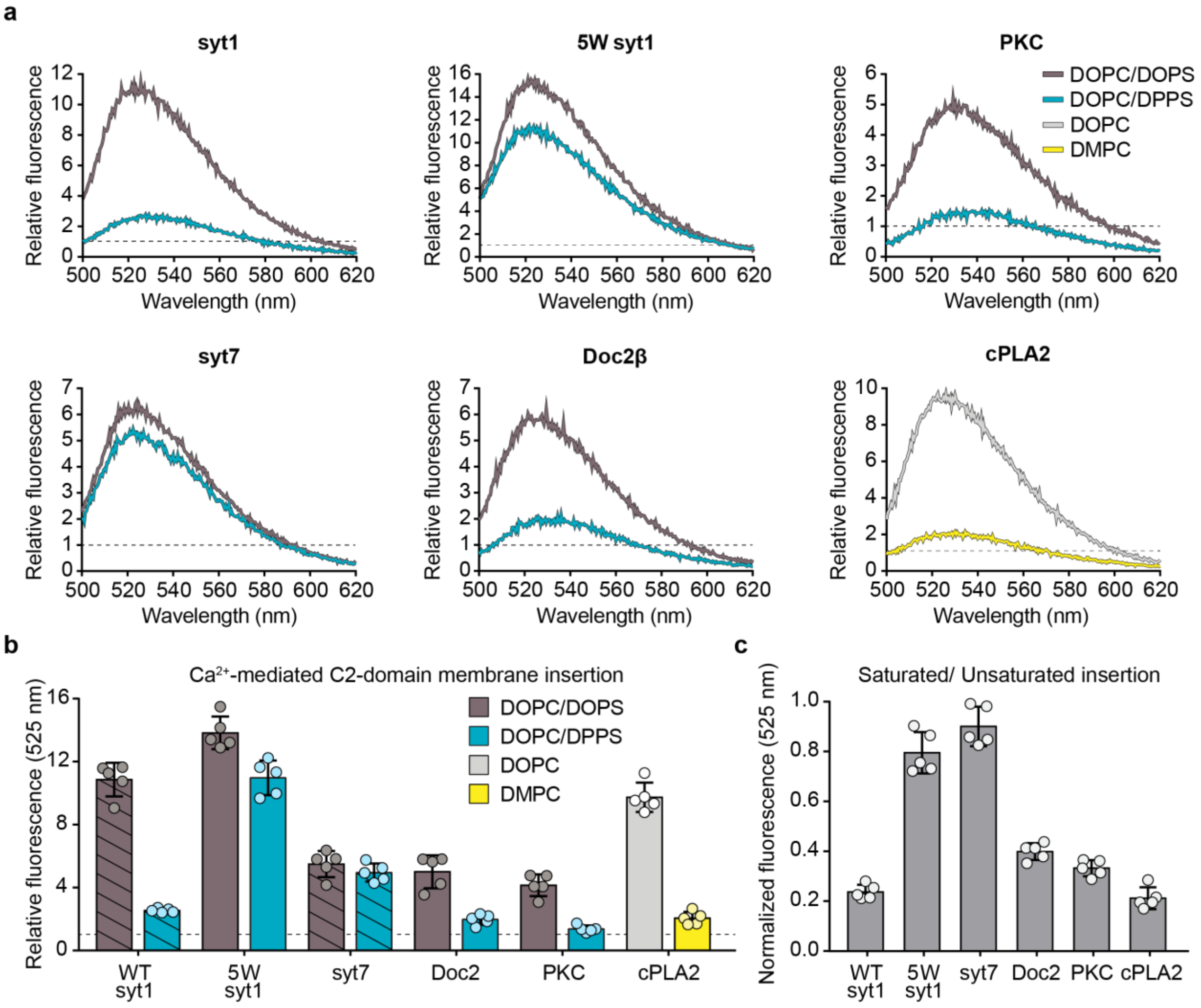

Supplementary Fig. 3. **Doc2β, PKC and cPLA2 fail to efficiently penetrate bilayers with saturated acyl chains. a** Representative fluorescence spectra from NBD labeled Doc2β, PKC and cPLA2 in the presence and absence of Ca^{2+}. The NBD fluorescence in EGTA is represented as a black dotted line. The Doc2β and PKC experiments were performed with liposomes composed of DOPC/DOPS or DOPC/DPPS (80:20); the cPLA2 experiments were performed with liposomes composed of DOPC or DMPC, due to the inherent lack of PS binding specificity. The syt1 and syt7 C2AB traces are reproduced from Fig. 1 for comparison. **b** Quantification of replicated experiments described in *panel a.* Replicates were performed five times on separate days with fresh lipid samples. The fluorescence intensity at 525 nm was extracted from each spectrum, normalized to the EGTA condition and plotted. The horizontal dotted line represents the normalized fluorescence intensity the EGTA conditions. The syt1 and syt7 data are reproduced from Fig. 1 for comparison and emphasized with diagonal lines. **c** Normalized NBD fluorescent intensities at 525 nm from data represented in *panel b*. The fluorescence in the saturated acyl chain conditions were divided by the condition containing all unsaturated acyl chains. Error bars represent standard error of the mean.

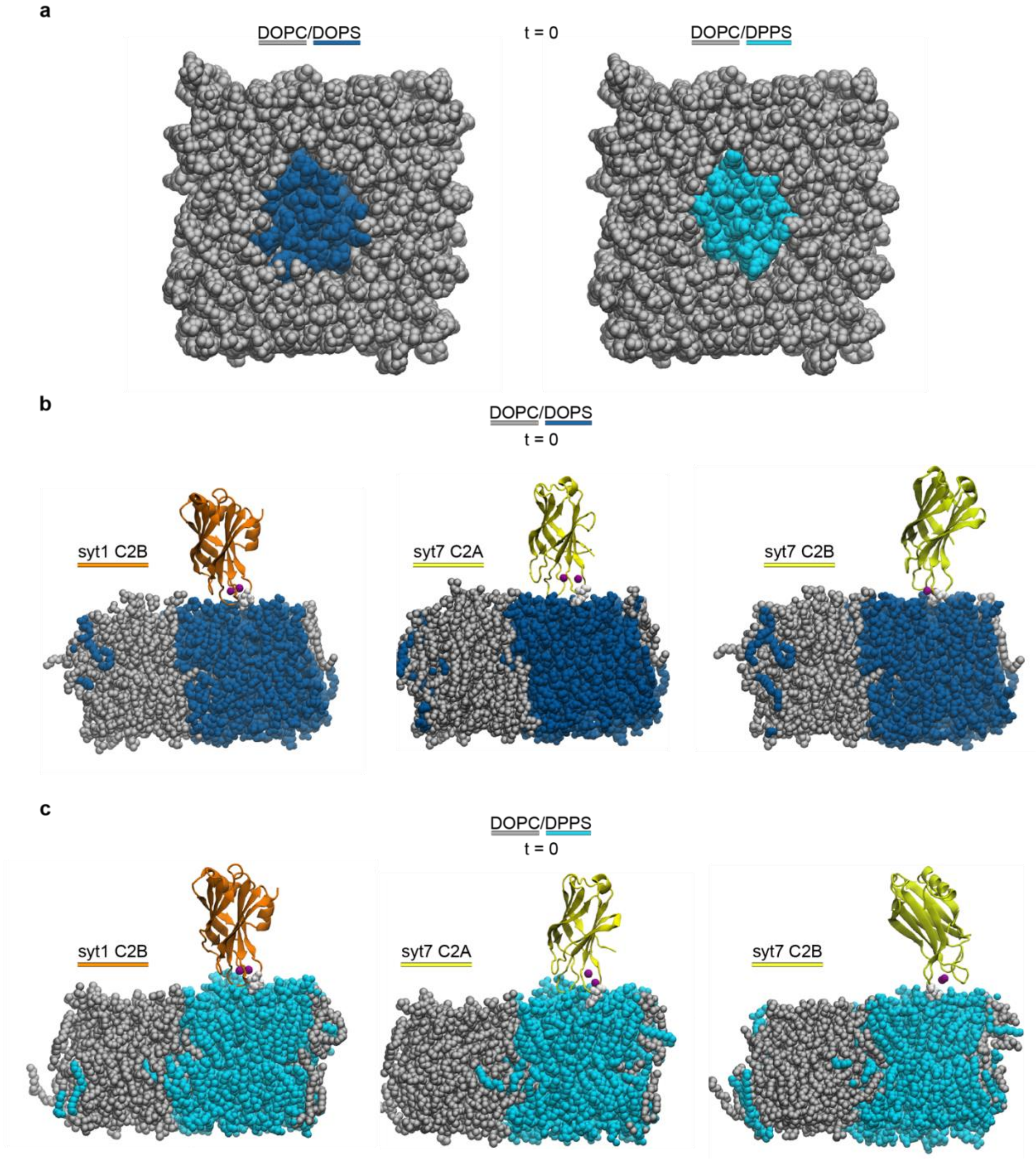

Supplementary Fig. 4. **Initial configurations of MD simulations. a** Time zero snapshots of MD simulations that examine the clustering behavior of DOPS (*left*) and DPPS (*right*) among DOPC lipids (grey). **b** Time zero snapshots of MD simulations comparing syt1 C2B (shown in orange) domain with syt7 C2A and C2B domains (yellow) positioned on bilayers containing 20% DOPS. **c** Time zero snapshots of MD simulations comparing syt1 C2B (shown in orange) domain with syt7 C2A and C2B domains (yellow) positioned on bilayers containing 20% DPPS..

Corresponding end-point snapshots for syt1 and syt7 C2B domains are found in Figure 2. The syt7 C2A end-point snapshots are shown in Supplementary Fig. 6.

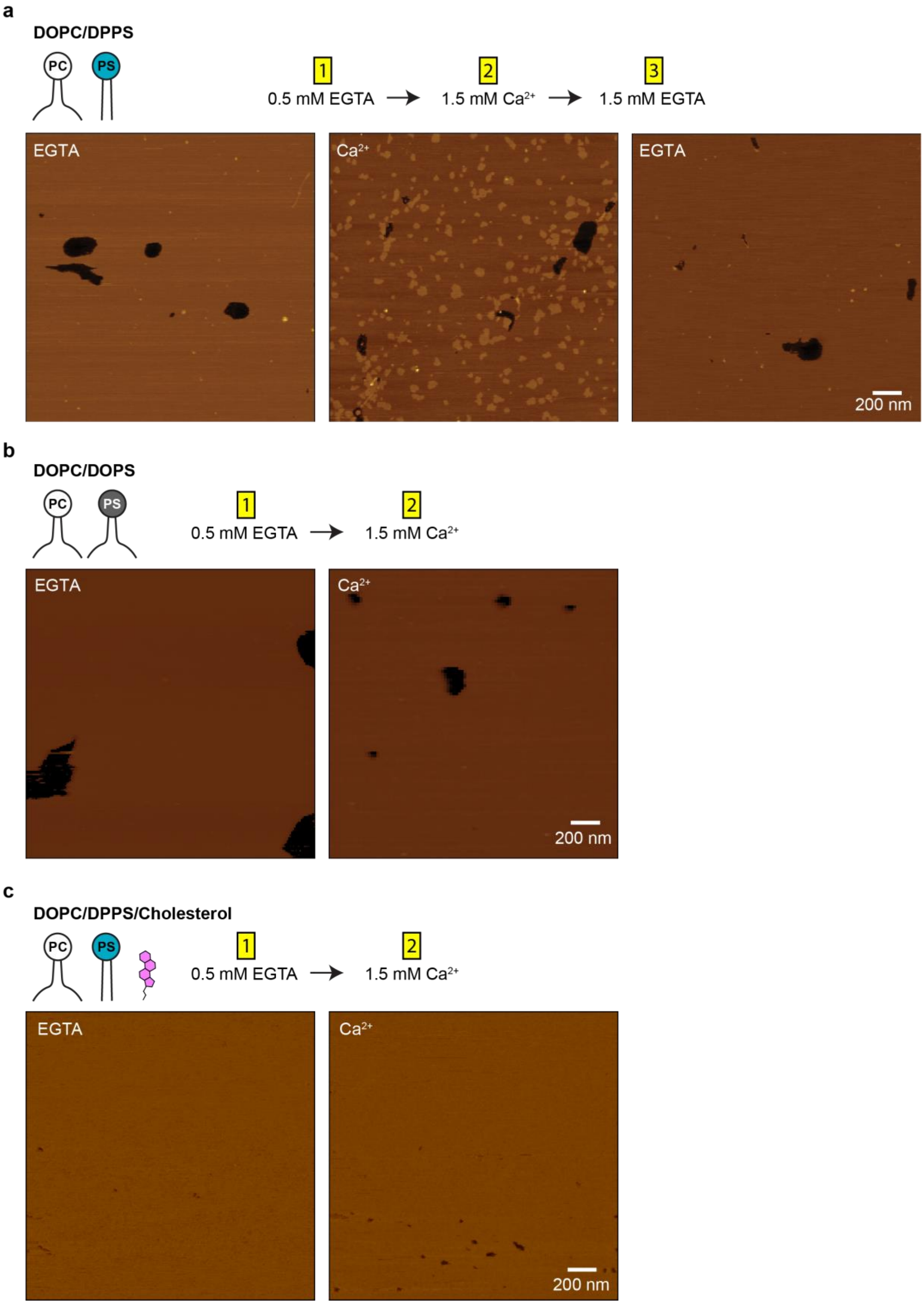

a
DOPC/DPPS
PC
PS
1
0.5 mM EGTA
2
1.5 mM Ca2+
3
1.5 mM EGTA
EGTA
Ca2+
EGTA
200 nm
b
DOPC/DOPS
PC
PS
1
0.5 mM EGTA
2
1.5 mM Ca2+
EGTA
Ca2+
200 nm
c
DOPC/DPPS/Cholesterol
PC
PS
1
0.5 mM EGTA
2
1.5 mM Ca2+
EGTA
Ca2+
200 nm

Supplementary Fig. 5. **Ca^{2+} causes DPPS clustering in lipid bilayers. a** AFM imaging of supported lipid bilayers on mica composed of DOPC/DPPS (80:20) with and without Ca^{2+}. **b** AFM imaging of supported lipid bilayers on mica composed of DOPC/DOPS (80:20) with and without Ca^{2+}. **c** AFM imaging of supported lipid bilayers on mica composed of DOPC/DPPS/cholesterol (56:14:30) with and without Ca^{2+}.

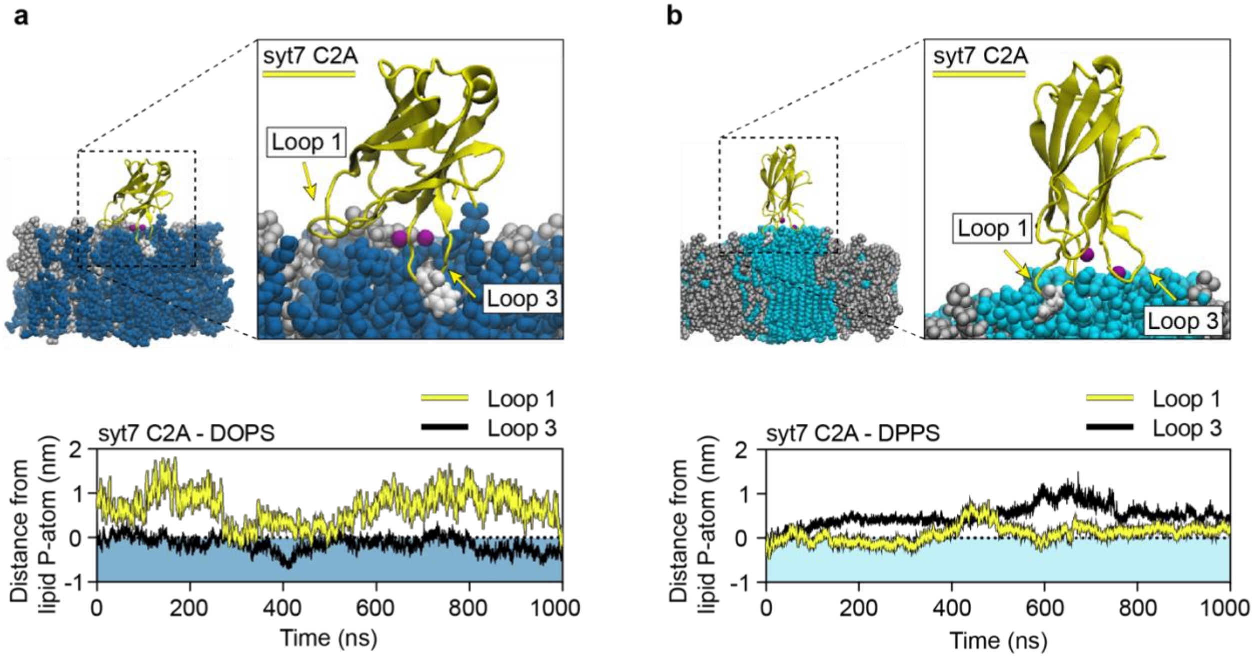

Supplementary Fig. 6. **Molecular dynamics simulations of syt7 C2A•membrane interactions. a** End point (1000 ns) MD simulations snapshot showing the syt7 (yellow) C2A domain interacting with a lipid bilayer composed of DOPC/DOPS. The loop residue that achieved the deepest depth of penetration, F229, is emphasized in white. **b** End point (1000 ns) MD simulations snapshot showing the syt7 (yellow) C2A domain interacting with a lipid bilayer composed of DOPC/DPPS. The loop residue that achieved the deepest depth of penetration, F167, is emphasized in white. Quantification of loop 1 and loop 3 depth from syt7 C2A into the DOPS-containing (blue shading) and DPPS-containing (cyan shading) bilayers are shown in the lower panels. In each case, the loop depth is normalized relative to the position of the lipid phosphate group, indicated by a horizontal dotted line.

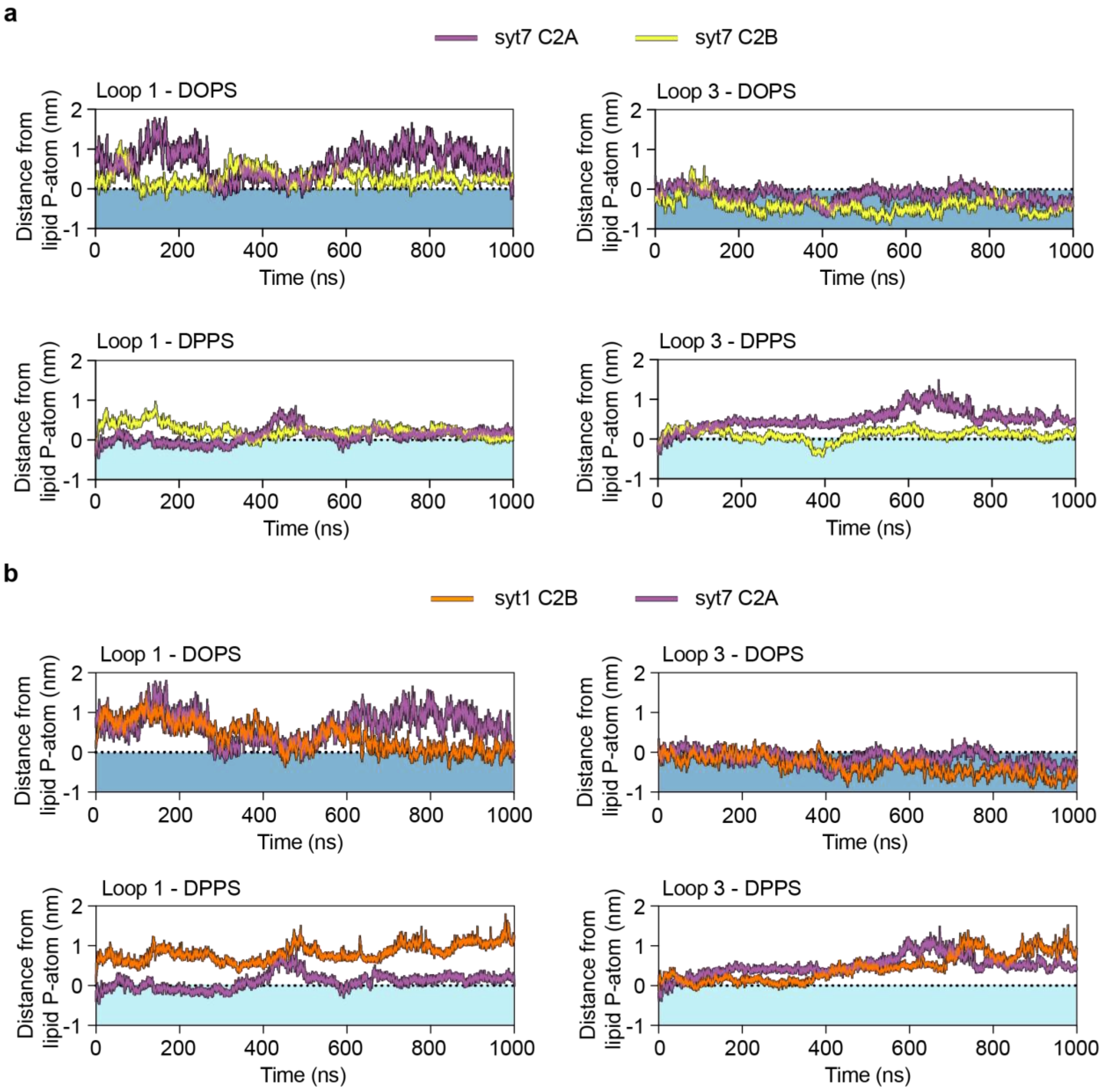

Supplementary Fig. 7. **MD simulations comparison of membrane penetration depth between syt7 C2A and syt1 or syt7 C2B domains. a** Quantification of loop 1 and loop 3 depth of syt7 C2A (purple) and syt7 C2B (yellow) domains into the DOPS-containing (blue shading) and DPPS-containing (cyan shading) bilayers. **b** Quantification of loop 1 and loop 3 depth of syt1 C2B (orange) and syt7 C2A (purple) domains into the DOPS-containing (blue shading) and DPPS-containing (cyan shading) bilayers. These data are re-plotted quantifications from Fig. 2 and Supplementary Fig. 6.

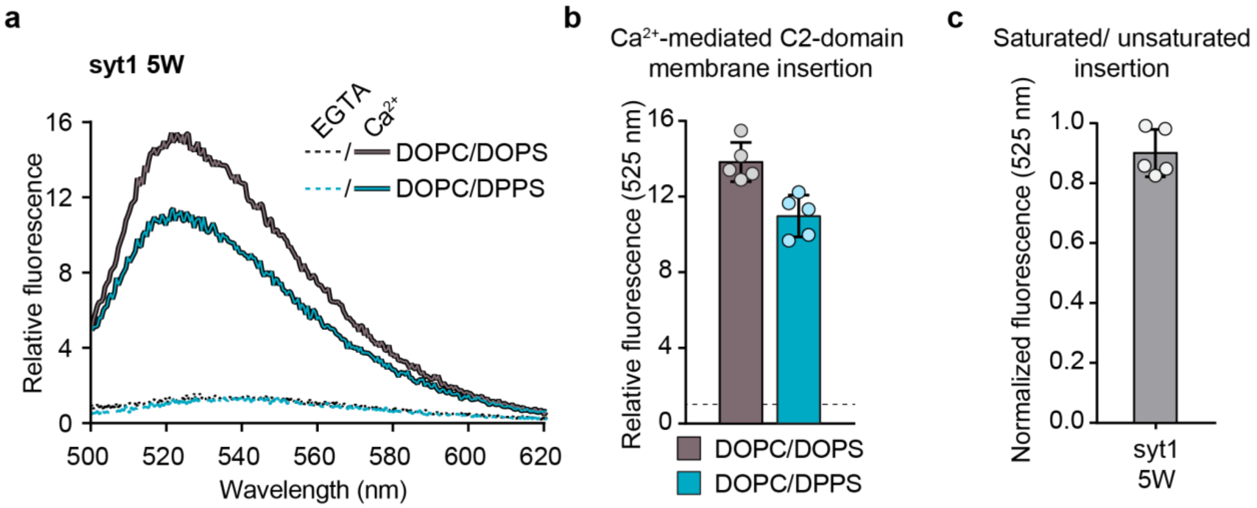

Supplementary Fig. 8. **Tryptophan substitutions in the penetration loops enables syt1 to penetrate bilayers that harbor DPPS. a** Representative fluorescence spectra of NBD-labelled syt1 5W C2AB in 0.2 mM EGTA (dotted lines) or 0.5 mM free Ca^{2+} (solid lines) and with liposomes composed of DOPC/DOPS or DOPC/DPPS (80:20). **b** Quantification of replicated experiments depicted in *panel a*. The normalized fluorescence intensity at 525 nm in Ca^{2+} is shown relative to the EGTA condition. **c** Normalized NBD fluorescent intensity at 525 nm from data represented in *panel b*. The fluorescence in the saturated acyl chain conditions were divided by the condition containing all unsaturated acyl chains. Each condition was repeated five times on different days using fresh materials. Error bars represent standard error of the mean.

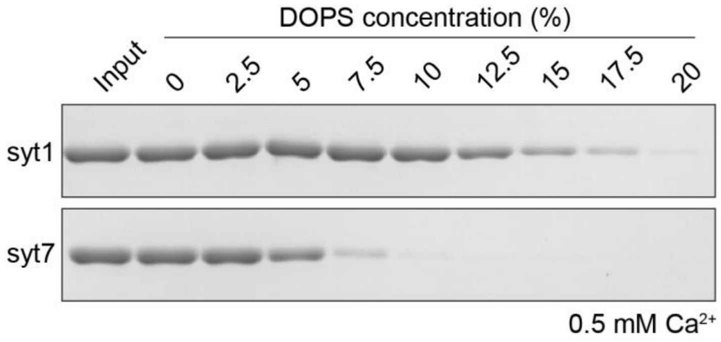

Supplementary Fig. 9. **Syt7 displays higher affinity for PS-bearing liposomes than syt1.** Representative Coomassie stained SDS-PAGE gel of a co-sedimentation assay (described in Fig. 4a) with syt1 and syt7 C2AB domains in the presence of Ca^{2+} and liposomes with increasing mole fractions of PS. Depletion of protein from the samples indicates binding to liposomes. Input refers to the protein-only control sample. The experiment was repeated on three separate occasions with consistent results.

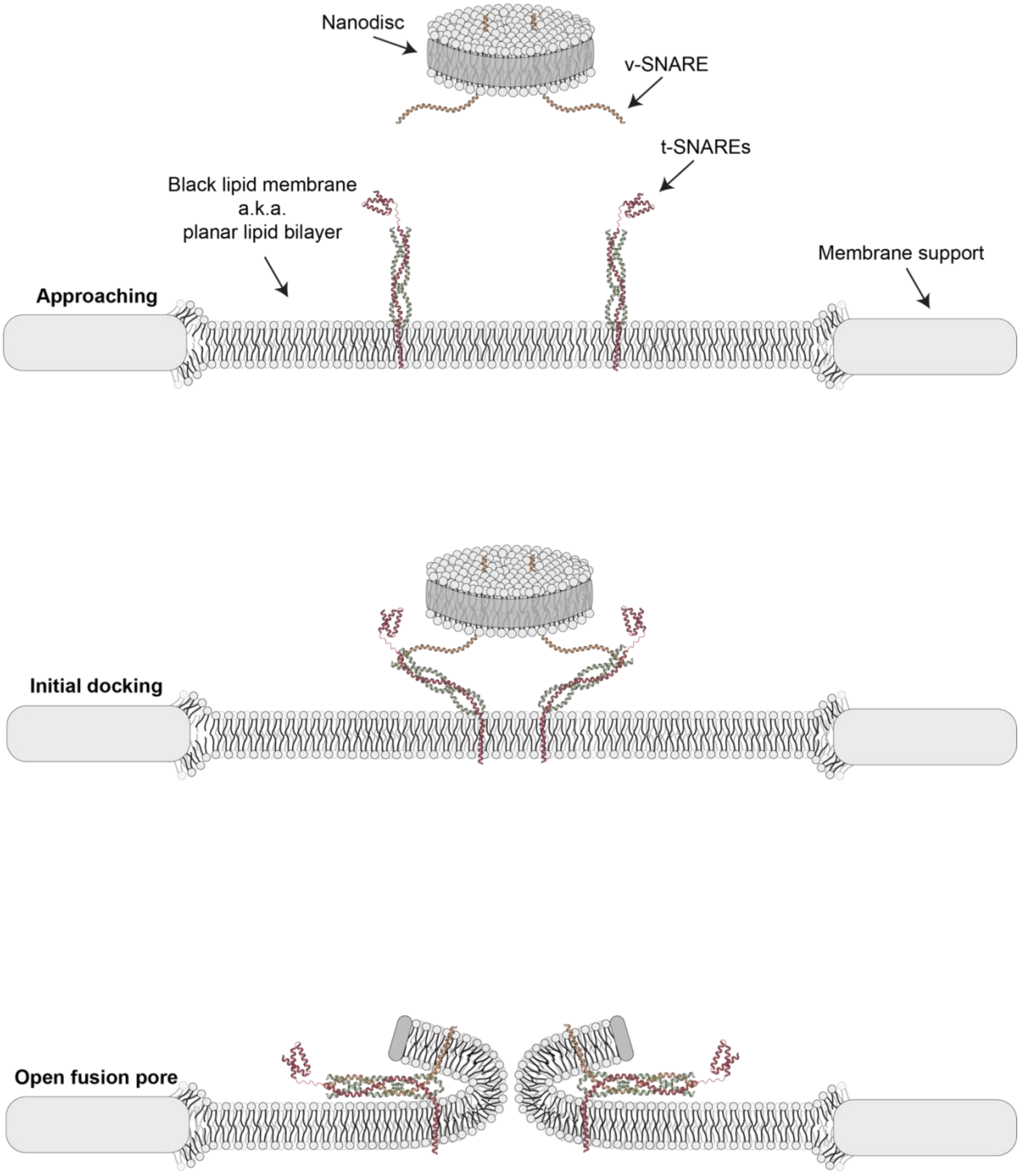

Supplementary Fig. 10. **Assembly steps for fusion pore formation during nanodisc-black lipid membrane electrophysiology experiments.** An illustration showing the sequence of events leading up to the formation of a fusion pore formed between a v-SNARE nanodisc and a t-SNARE black lipid membrane.

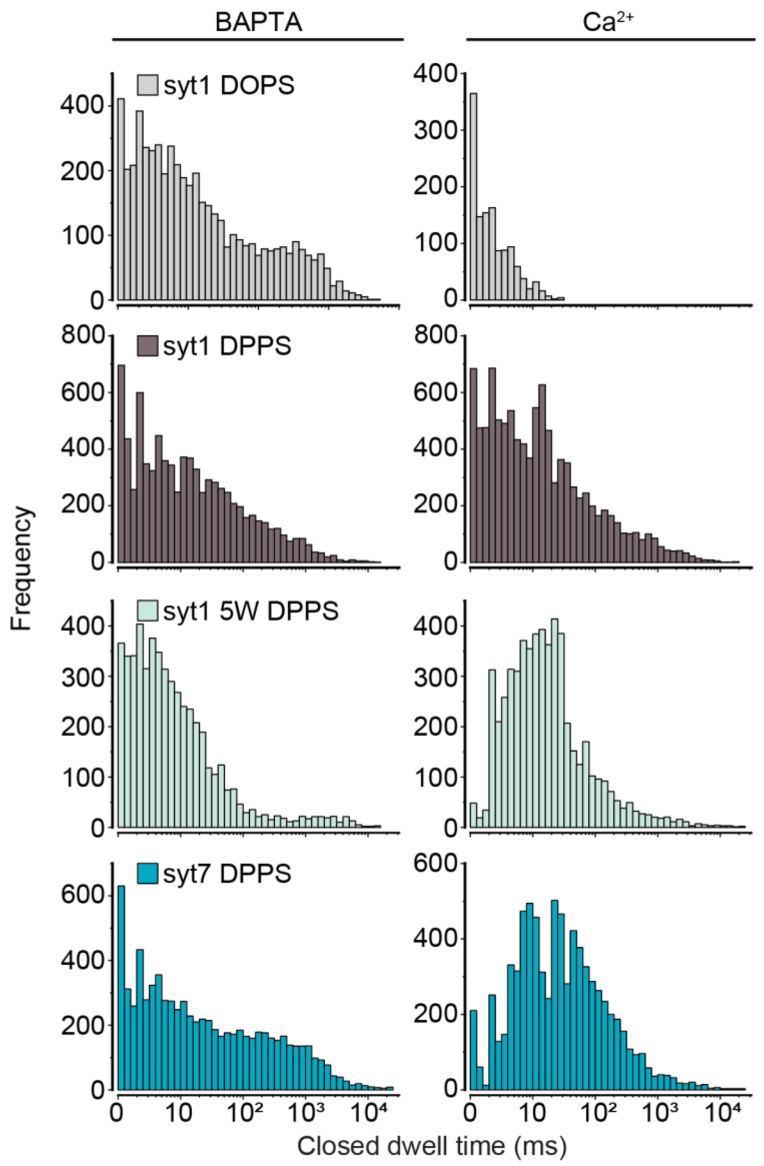

Supplementary Fig. 11. **In the presence of Ca^{2+} and ND_3-syt1 5W or ND_3-syt7, SNARE-mediated fusion pores exhibit long closed dwell times when the BLM harbors DOPC/DPPS.** Closed dwell time distributions generated and pooled from each of the indicated ND-BLM conditions, in 0.5 mM BAPTA or 0.5 mM free Ca^{2+}. Associated data are found in Fig. 5.

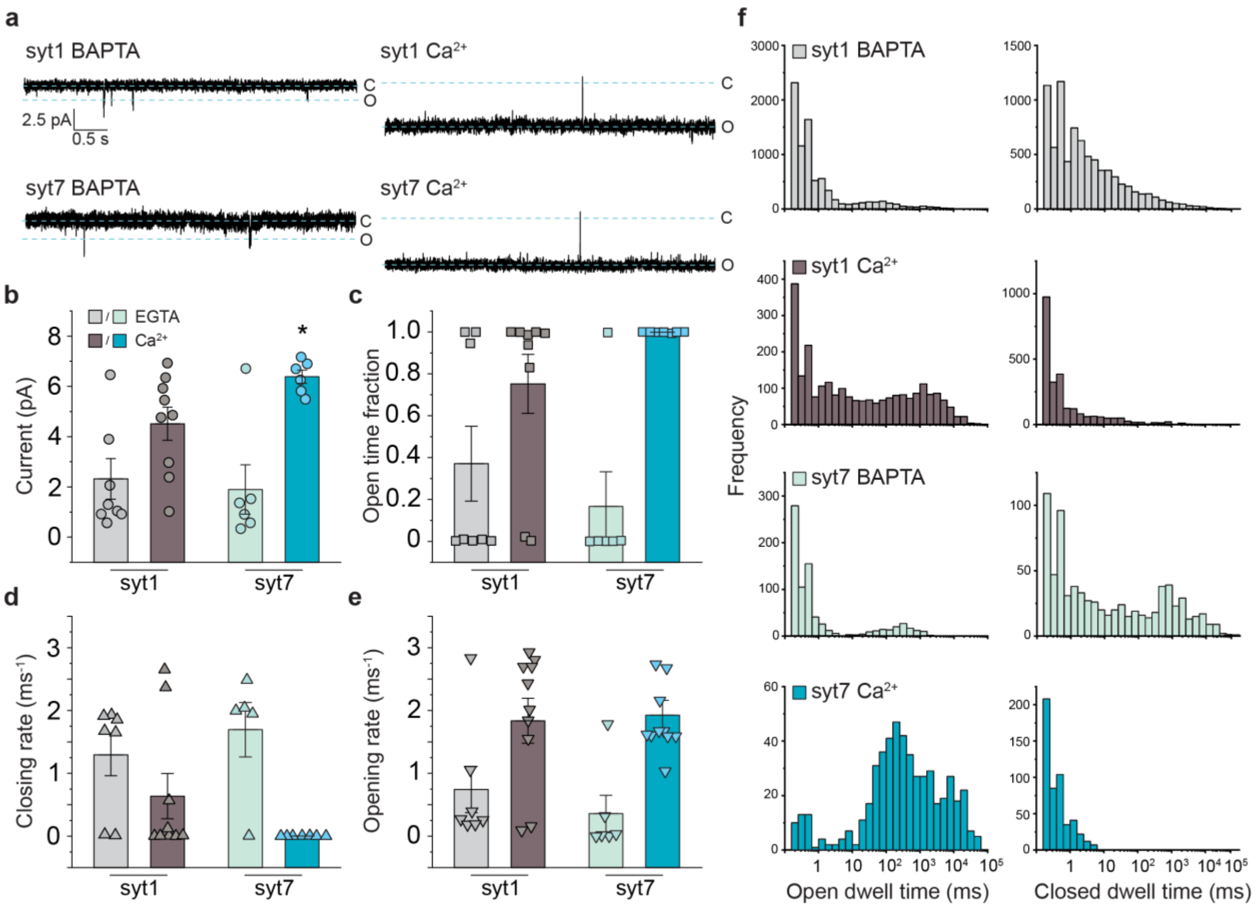

Supplementary Fig. 12. **The differential effects of syt1 and syt7 on the properties of fusion pores are subtle in membranes containing DOPS.** Results of ND-BLM experiments performed using classical BLM methodology with membranes containing 20% DOPS. **a** Representative ND-BLM traces from fusion pores formed by ND_3-syt1 and ND_3-syt7 in 0.5 mM BAPTA or 0.5 mM free Ca^{2+}. **b** Quantification of the current passing through ND-BLM fusion pores under the indicated conditions. **c** Quantification of the fraction of time that ND-BLM fusion pores remaFig. 3ined in the open state under the indicated conditions. **d** Closing rates of ND-BLM fusion pores derived from the indicated open dwell time analyses. **e** Opening rates of ND-BLM fusion pores derived from the indicated closed dwell time analyses. **f** Open and closed dwell time distributions from the indicated ND-BLM fusion pore conditions. The data from each replicated condition are pooled. Each condition was repeated six times on different days using fresh materials. Error bars represent standard error of the mean. The asterisk (*) in *panel b* indicates a significant difference between syt1 and syt7 in the 0.5 mM free Ca^{2+} condition was determined via Student's t-test. All other comparisons between syt1 and syt7 throughout the figure are not significantly different.
