# Supplementary material for: Synaptotagmin-7 outperforms synaptotagmin-1 to promote the formation of large, stable fusion pores via robust membrane penetration
Source: Nat Commun. 2023 Nov 27;14:7761. doi: 10.1038/s41467-023-42497-8 (PMC10681989; doi:10.1038/s41467-023-42497-8)
Supplement: Supplementary file 3 — Description of Additional Supplementary files [file 41467_2023_42497_MOESM3_ESM.pdf]

Description of additional Supplementary files

File name: Supplementary movie 1

Description: MD simulation of DOPS clustering within a DOPC bilayer. A representative MD simulation showing a cluster of DOPS (blue) lipids within a DOPC bilayer (grey). The DOPS was placed at the center of the bilayer and allowed to equilibrate with the DOPC for 450 ns.

File name: Supplementary movie 2

Description: MD simulation of DPPS clustering within a DOPC bilayer. A representative MD simulation showing a cluster of DPPS (cyan) lipids within a DOPC bilayer (grey). The DPPS was placed at the center of the bilayer and allowed to equilibrate with the DOPC for 450 ns.
