# Supplementary material for: Synaptotagmin-7 outperforms synaptotagmin-1 to promote the formation of large, stable fusion pores via robust membrane penetration
Source: Nat Commun. 2023 Nov 27;14:7761. doi: 10.1038/s41467-023-42497-8 (PMC10681989; doi:10.1038/s41467-023-42497-8)
Supplement: Supplementary file 6 — Reporting Summary [file 41467_2023_42497_MOESM6_ESM.pdf]

nature portfolio

Corresponding author(s): Edwin Chapman

Last updated by author(s): Oct 2, 2023

Reporting Summary

Nature Portfolio wishes to improve the reproducibility of the work that we publish. This form provides structure for consistency and transparency in reporting. For further information on Nature Portfolio policies, see our Editorial Policies and the Editorial Policy Checklist.

Statistics

For all statistical analyses, confirm that the following items are present in the figure legend, table legend, main text, or Methods section.

n/a	Confirmed	
☐	☒	The exact sample size (*n*) for each experimental group/condition, given as a discrete number and unit of measurement
☐	☒	A statement on whether measurements were taken from distinct samples or whether the same sample was measured repeatedly
☐	☒	The statistical test(s) used AND whether they are one- or two-sided<br>*Only common tests should be described solely by name; describe more complex techniques in the Methods section.*
☒	☐	A description of all covariates tested
☒	☐	A description of any assumptions or corrections, such as tests of normality and adjustment for multiple comparisons
☐	☒	A full description of the statistical parameters including central tendency (e.g. means) or other basic estimates (e.g. regression coefficient) AND variation (e.g. standard deviation) or associated estimates of uncertainty (e.g. confidence intervals)
☐	☒	For null hypothesis testing, the test statistic (e.g. *F*, *t*, *r*) with confidence intervals, effect sizes, degrees of freedom and *P* value noted<br>*Give P values as exact values whenever suitable.*
☒	☐	For Bayesian analysis, information on the choice of priors and Markov chain Monte Carlo settings
☒	☐	For hierarchical and complex designs, identification of the appropriate level for tests and full reporting of outcomes
☒	☐	Estimates of effect sizes (e.g. Cohen's *d*, Pearson's *r*), indicating how they were calculated

Our web collection on statistics for biologists contains articles on many of the points above.

Software and code

Policy information about availability of computer code

Data collection	Data was acquired using Felix GX, Applied Photophysics Pro-Data SX, pClamp 11 and CHARMM-GUI software.
Data analysis	data analysis was performed using Graphpad Prism v9 and ImageJ2

For manuscripts utilizing custom algorithms or software that are central to the research but not yet described in published literature, software must be made available to editors and reviewers. We strongly encourage code deposition in a community repository (e.g. GitHub). See the Nature Portfolio guidelines for submitting code & software for further information.

Data

Policy information about availability of data

All manuscripts must include a data availability statement. This statement should provide the following information, where applicable:

- Accession codes, unique identifiers, or web links for publicly available datasets
- A description of any restrictions on data availability
- For clinical datasets or third party data, please ensure that the statement adheres to our policy

Source data are provided with this paper. All data presented in this manuscript can be found in the accompanying source data file. Structures used in the molecular dynamics simulations were acquired from RCSB.org.

Research involving human participants, their data, or biological material

Policy information about studies with human participants or human data. See also policy information about sex, gender (identity/presentation), and sexual orientation and race, ethnicity and racism.

Reporting on sex and gender	This study is a combination of in vitro and theoretical work. No gender related information is relevant.
Reporting on race, ethnicity, or other socially relevant groupings	This study is a combination of in vitro and theoretical work. No race related information is relevant.
Population characteristics	This study is a combination of in vitro and theoretical work. No population characteristics are relevant.
Recruitment	This study is a combination of in vitro and theoretical work. No recruitment related information is relevant.
Ethics oversight	This study is a combination of in vitro and theoretical work. No Ethics related information is relevant.

Note that full information on the approval of the study protocol must also be provided in the manuscript.

Field-specific reporting

Please select the one below that is the best fit for your research. If you are not sure, read the appropriate sections before making your selection.

[x] Life sciences [ ] Behavioural & social sciences [ ] Ecological, evolutionary & environmental sciences

For a reference copy of the document with all sections, see nature.com/documents/nr-reporting-summary-flat.pdf

Life sciences study design

All studies must disclose on these points even when the disclosure is negative.

Sample size	No statistical methods were used to predetermine sample sizes. Sample sizes were determined based on the variability of the results in each experiment. The sample sizes used in each experiment were sufficient to show consistent trends in the data that could be analyzed.
Data exclusions	All acquired data are included in the manuscript.
Replication	All data presented in this manuscript were repeated at a minimum of three individual times on separate days with unique materials. All replicated experiments are included in the analysis and source data file.
Randomization	No formal randomization was performed, but each dataset was acquired randomly and discontinuously that controlled for systematic error.
Blinding	No formal blinding was performed. The data were acquired and analyzed without outcome bias and all data were included in the analysis, regardless of the result.

Reporting for specific materials, systems and methods

We require information from authors about some types of materials, experimental systems and methods used in many studies. Here, indicate whether each material, system or method listed is relevant to your study. If you are not sure if a list item applies to your research, read the appropriate section before selecting a response.

Materials & experimental systems

n/a	Involved in the study
[x]	[ ] Antibodies
[x]	[ ] Eukaryotic cell lines
[x]	[ ] Palaeontology and archaeology
[x]	[ ] Animals and other organisms
[x]	[ ] Clinical data
[x]	[ ] Dual use research of concern
[x]	[ ] Plants

Methods

n/a	Involved in the study
[x]	[ ] ChIP-seq
[x]	[ ] Flow cytometry
[x]	[ ] MRI-based neuroimaging
