# Supplementary material for: Synaptotagmin-7 outperforms synaptotagmin-1 to promote the formation of large, stable fusion pores via robust membrane penetration
Source: Nat Commun. 2023 Nov 27;14:7761. doi: 10.1038/s41467-023-42497-8 (PMC10681989; doi:10.1038/s41467-023-42497-8)
Supplement: Supplementary file 2 — Peer Review File [file 41467_2023_42497_MOESM2_ESM.pdf]

nature portfolio

Peer Review File

Synaptotagmin-7 outperforms synaptotagmin-1 to promote the formation of large, stable fusion pores via robust membrane penetration

Open Access This file is licensed under a Creative Commons Attribution 4.0 International License, which permits use, sharing, adaptation, distribution and reproduction in any medium or format, as long as you give appropriate credit to the original author(s) and the source, provide a link to the Creative Commons license, and indicate if changes were made. In the cases where the authors are anonymous, such as is the case for the reports of anonymous peer reviewers, author attribution should be to 'Anonymous Referee' followed by a clear attribution to the source work. The images or other third party material in this file are included in the article's Creative Commons license, unless indicated otherwise in a credit line to the material. If material is not included in the article's Creative Commons license and your intended use is not permitted by statutory regulation or exceeds the permitted use, you will need to obtain permission directly from the copyright holder. To view a copy of this license, visit http://creativecommons.org/licenses/by/4.0/.

REVIEWER COMMENTS

Reviewer #1 (Remarks to the Author):

This is an interesting manuscript concerning the membrane penetration properties of the tandem C2AB domains in Syt1 and Sty7 and their relevance to their membrane binding and fusion pore dynamics in calcium-triggered SNARE-mediated membrane fusion. It was found that Syt7 C2AB domain binds to the membrane containing ordered DPPS lipids by partially penetrating the membrane, while Syt1 C2AB domain fails to bind and partially penetrate the same membrane. Replacing the DPPS lipids with the disordered DOPS lipids or adding cholesterol into the membrane rescues Syt1 C2AB binding. These conclusions are supported by molecular dynamics simulations. The authors further found that the hydrophobic residues in the membrane binding loops of C2AB domains greatly contribute to their binding to the DPPS-containing membranes. Electric recording of single fusion pores suggests that membrane penetration is required for Syt1 and Syt7 mediated fusion pore opening and dilation. Overall, this manu reports novel and interesting results on the mechanism of C2AB binding to membranes and their function in calcium-triggered and SNARE-mediated membrane fusion, which represent important progress in the membrane fusion field. The manu is well-written, and the experiments are carefully designed and conducted, with beautiful data that support the major conclusions. Therefore, the manu is highly recommended for publication in Nature Communications, with minor revisions to address the following comments:
1. The authors appear to focus on the C2B domain in both Syt1 and Syt7. However, it is reported that Syt7 C2A domain dominates the membrane binding (Voleti et al., PNAS 2017). What is the role of C2A in membrane penetration in a saturated lipid bilayer?
2. How are saturated lipids distributed in cell membranes? Are their distributions correlated with the specific functions of syt1 and sy7 in synchronous and asynchronous exocytosis?
3. In Fig. 2C, it will be helpful to highlight loop 1 and loop 3.
4. In Fig. 4A, it is not clear why LUVs precipitate. What buffer or reagents are put inside the LUVs that make them heavier in water?
5. In Fig. 5, it is not clear how the beautiful current trajectories are analyzed to derive the lifetimes of open and closed states and their probabilities. In principle, hidden Markov modeling can be used to unambiguously extract these properties (e.g., Zhang, Y.L., Jiao, J. & Rebane, A.A. Hidden Markov modeling with detailed balance and its application to single protein folding Biophys. J. 111, 2110-2124, 2016). These analyses will better support the PORE DILATION mediated by Syt1 and Syt7. In Fig. 5C, what's the current labeled on the y-axis? Is it the average current or the current of the open pore state? In Fig. 5D, the opening probabilities of Syt1 and Syt7 with DPPS are close to or slightly greater than 0.5, but the corresponding current traces in Fig. 5C show significantly higher opening probabilities. It will be better to show typical current traces.
6. A reference may be needed for the sentence in lines 78-80.

Yongli Zhang and Jie Yang (Postdoc in the Zhang lab)

Reviewer #2 (Remarks to the Author):

Synaptotagmin 7 outperforms synaptotagmin 1 to promote the formation of large, stable fusion pores via robust membrane penetration

By Kevin C. Courtney, submitted to Nature Communications

The manuscript describes two important components in synaptic membrane fusion, the synaptotagmin 1 and 7. It illuminates how the distinct properties of the two proteins influence the pore formation related to membrane fusion. A combination of different techniques enables to identify the influence of membrane lipids thus entangling the influence of protein-protein interactions vs protein membrane interactions. A broad range of methods is used, fluorescence labelling to detect membrane penetration and membrane pore formation by a novel variant of black lipid membranes. The importance of

synaptic vesicle fusion and the problems in understanding the mechanism makes the manuscript extremely relevant.
For other fields the combination of techniques and the novel information on this particular part of the SNARE system could extend our knowledge, further in membrane protein fields the approach is also highly innovative and interesting.
The manuscript is clearly written and the methods used are explained very well.
Some questions arise, however:
1. In the paragraph starting at line 201 the effect of cholesterol on syt1 penetration is described. At 30 % cholesterol complete membrane penetration is observed, however that seems like a very high cholesterol concentration under normal circumstances. Could the authors describe the physiological relevance of such high concentrations?
2. line 251: 5 W residues are substituted into the membrane penetration loops of syt1. It is argued that these residues make the loop hydrophobic and the observed effects are explained in terms of deeper membrane penetration because of increased hydrophobicity. There is quite a bit of literature that describes W as a lipid headgroup anchor, specifically localizing W in the headgroup regions of the lipid. Would that be a different explanation for the observed effects? Perhaps the manuscript should comment on this point or give arguments why the hydrophobicity is dominant in the case of the syt1 5W.
3. on line 312: 'nanodisc-black lipid membrane (ND-BLM)'. I find the term nano-disc in this context confusing: Usually nanodisk refers to nano-meter sized, solubilized planar membranes that are stabilized by a protein or polymer. In the context of the manuscript it seems that the expression nanodisc derives purely from the hole-size of the BLM cup. Could the authors explain? Or is that standard usage in the field of BLM membrane electric measurements?
After taking into account the above minor points this manuscript is a very nice contribution, fit for publication in Nature Communications.

Reviewer #3 (Remarks to the Author):

In this report, the authors use biochemical and computational methods to demonstrate that the membrane interaction/penetration properties of the Synaptotagmin (Syt)-7 C2B domain is more robust than the Syt1 C2B domain. Using targeted mutations, they identify that the hydrophobic properties of the C2B acyl loops is a critical determinant of membrane penetrating activity. They further employ nanodisc-black lipid membrane (BLM) electrophysiology to correlate the membrane penetrating properties to the Syt1/7 ability to regulate fusion pores. This is a timely and well-written manuscript and provides crucial biochemical/molecular insights into the Syt7 protein. However, additional data is needed to understand the physiological/functional relevance of these findings.

1) Considering that the observed difference in membrane interaction activity of Syt1 and Syt7 is largely abrogated in the presence of physiological level (~30%) cholesterol in the lipid bilayer, it is unclear if the ascribed difference is physiologically and functionally relevant. It is likely that the Syt1 and Syt7 C2B domains might act very similarly under native cellular conditions especially since the authors do not provide any data on the behavior of Syt7 under cholesterol-containing conditions.

2) The authors have not included data on the effect of Syt7 on fusion pores formed with DOPS containing BLM. This is critical as the pore regulatory properties of Syt7 (e.g., current, open time fraction, opening rate shown in Figure 5C, D, F) under the DPPS condition are lower than that of Syt1 under DOPS conditions. Since Syt7 acts similarly under both DOPS and DPPS conditions, it stands to reason that Syt7 might actually be less efficient than Syt1 to stabilize the fusion pore under DOPS conditions.

3) The manuscript is focused on the C2B domain of the Syt isoforms. While the C2B domain is critical for the Syt1 function, it is well-established that the Syt7 C2A domain is crucial to its function. Thus, it might be important to understand how the C2A domain of Syt7 compares to the Syt1 C2B domain.

Synaptotagmin 7 outperforms synaptotagmin 1 to promote the formation of large, stable fusion pores via robust membrane penetration - Courtney et al. **(NCOMMS-23-03354)**

Response to reviewer comments:

Reviewer #1 (Remarks to the Author):

This is an interesting manuscript concerning the membrane penetration properties of the tandem C2AB domains in Syt1 and Sty7 and their relevance to their membrane binding and fusion pore dynamics in calcium-triggered SNARE-mediated membrane fusion. It was found that Syt7 C2AB domain binds to the membrane containing ordered DPPS lipids by partially penetrating the membrane, while Syt1 C2AB domain fails to bind and partially penetrate the same membrane. Replacing the DPPS lipids with the disordered DOPS lipids or adding cholesterol into the membrane rescues Syt1 C2AB binding. These conclusions are supported by molecular dynamics simulations. The authors further found that the hydrophobic residues in the membrane binding loops of C2AB domains greatly contribute to their binding to the DPPS-containing membranes. Electric recording of single fusion pores suggests that membrane penetration is required for Syt1 and Syt7 mediated fusion pore opening and dilation. Overall, this manu reports novel and interesting results on the mechanism of C2AB binding to membranes and their function in calcium-triggered and SNARE-mediated membrane fusion, which represent important progress in the membrane fusion field. The manu is well-written, and the experiments are carefully designed and conducted, with beautiful data that support the major conclusions. Therefore, the manu is highly recommended for publication in Nature Communications, with minor revisions to address the following comments:

1. The authors appear to focus on the C2B domain in both Syt1 and Syt7. However, it is reported that Syt7 C2A domain dominates the membrane binding (Voleti et al., PNAS 2017). What is the role of C2A in membrane penetration in a saturated lipid bilayer?

This is an important comment that was shared by reviewers 1 and 3. The reviewers are correct to point out that aspects of our original manuscript compared membrane interactions of the syt1 and syt7 C2B domains. Specifically, the membrane penetration experiments in Fig. 1 placed the NBD reporter on the third loop of the C2B portion of each C2AB domain and the MD simulations shown in Fig. 2 only modeled the C2B domains. The remainder of the comparisons of syt1 and syt7 in our study assayed the C2AB domains or full-length proteins, largely indiscriminate of the individual C2-domains. The sole exception to this was in Fig. 4g and Fig. 4i, where we tested syt7 C2AB mutants with reduced loop hydrophobicity in either the C2A or C2B domains (syt7 $C2_{2A}B$ and syt7 $C2AB_{2A}$). These experiments support the importance of the C2A domain as we found that reduced hydrophobicity in syt7 C2A had significant impact on membrane binding. Reducing loop hydrophobicity in the C2B domain however, had no discernable effect. A reproduction of these data panels is shown here:

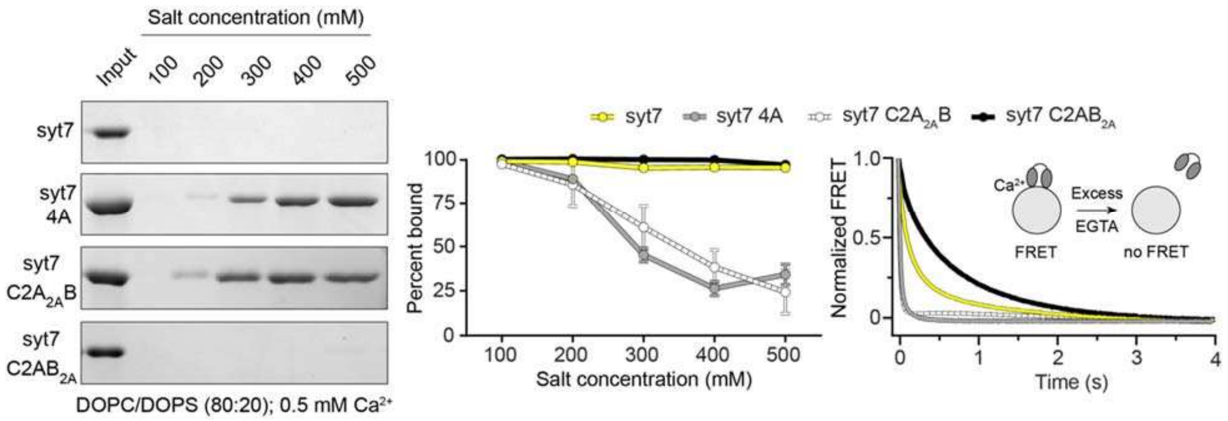

Revision Figure 1 – Reproduction of data panels from Fig. 4.

In designing this study, we found it logical to begin comparing syt1 and syt7 by monitoring their interactions with membranes at the same relative positions, in their 3-dimensional structures, for Figs. 1 and 2 (C2B, loop 3). However, the reviewers raise an interesting question that was not specifically addressed in our original manuscript. Namely, how does the C2A domain of syt7 perform against a DPPS-containing membrane? To directly address this issue, we generated new computational and experimental datasets to assess syt7 C2A-membrane interactions as a complement our original datasets.

Computationally, we generated a new set of MD simulations of the syt7 C2A domain interacting with membranes composed of DOPC/DOPS (80:20) or DOPC/DPPS (80:20); these simulations were carried out using the same parameters as our original computational efforts in Fig. 2. The C2A domain was placed at the membrane surface and allowed to equilibrate for 1000 ns. As expected, we found that the syt7 C2A domain was able to efficiently penetrate the bilayer containing DOPS. Interestingly, loop 3 of the syt7 C2A domain dominated the DOPS-membrane interaction, causing the C2A domain to tilt, restricting the depth of loop 1 penetration. When examining the interaction of syt7 C2A with the DPPS bilayer, our analysis again showed membrane penetration, which is in sharp contrast to the syt1 C2B domain. Loop 1 of the syt7 C2A domain penetrated well to the depth of lipid phosphate groups, while loop 3 penetration was on average, 0.5 nm above the phosphate layer engaging in interactions with the lipid head groups. An illustration of these new MD simulations is shown below (and these finding are also shown in Supplementary Fig. 6 of our revised manuscript).

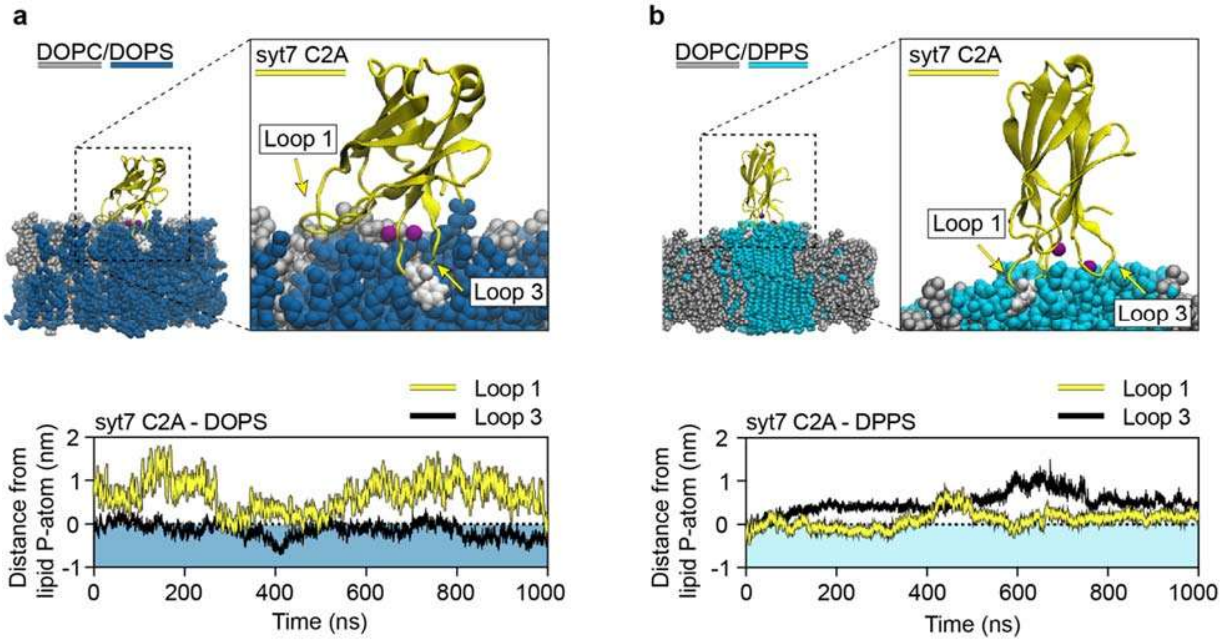

Revision Figure 2 (Revised manuscript Supplementary Fig. 6) – MD simulations of the syt7 C2A domain interacting with lipid bilayers containing DOPS or DPPS. A new set of MD simulations with syt7 C2A were performed to complement our original syt1 and syt7 C2B datasets.

With this new set of syt7 C2A MD simulations, we were then able to make additional pairwise comparisons. We generated a new supplementary figure (now Supplementary Fig. 7), shown below, that compares loop 1 and 3 penetration depth between syt7 C2A and C2B domains, as well as the syt7 C2A domain with the C2B domain of syt1. These additional pairwise comparisons show that each C2-domain that we tested efficiently penetrates DOPS membranes; only loop 1 of syt7 C2A largely remains on the DOPS membrane surface, due to tilting of the C2-domain.

In DPPS, both syt7 C2-domains exhibited similar membrane penetration. The penetration depth of loop 1 was nearly identical for both syt7 C2-domains, while loop 3 of C2A was slightly less than syt7 C2B. When comparing syt1 C2B with syt7 C2A in DPPS, a clear distinction is observed for loop 1; syt7 C2A is superior as syt1 C2B fails to penetrate. In contrast, loop 3 of syt1 C2B and syt7 C2A are more similar. Together, these additional MD simulations demonstrate that both C2-domains of syt7 contribute to efficient penetration into DPPS membranes.

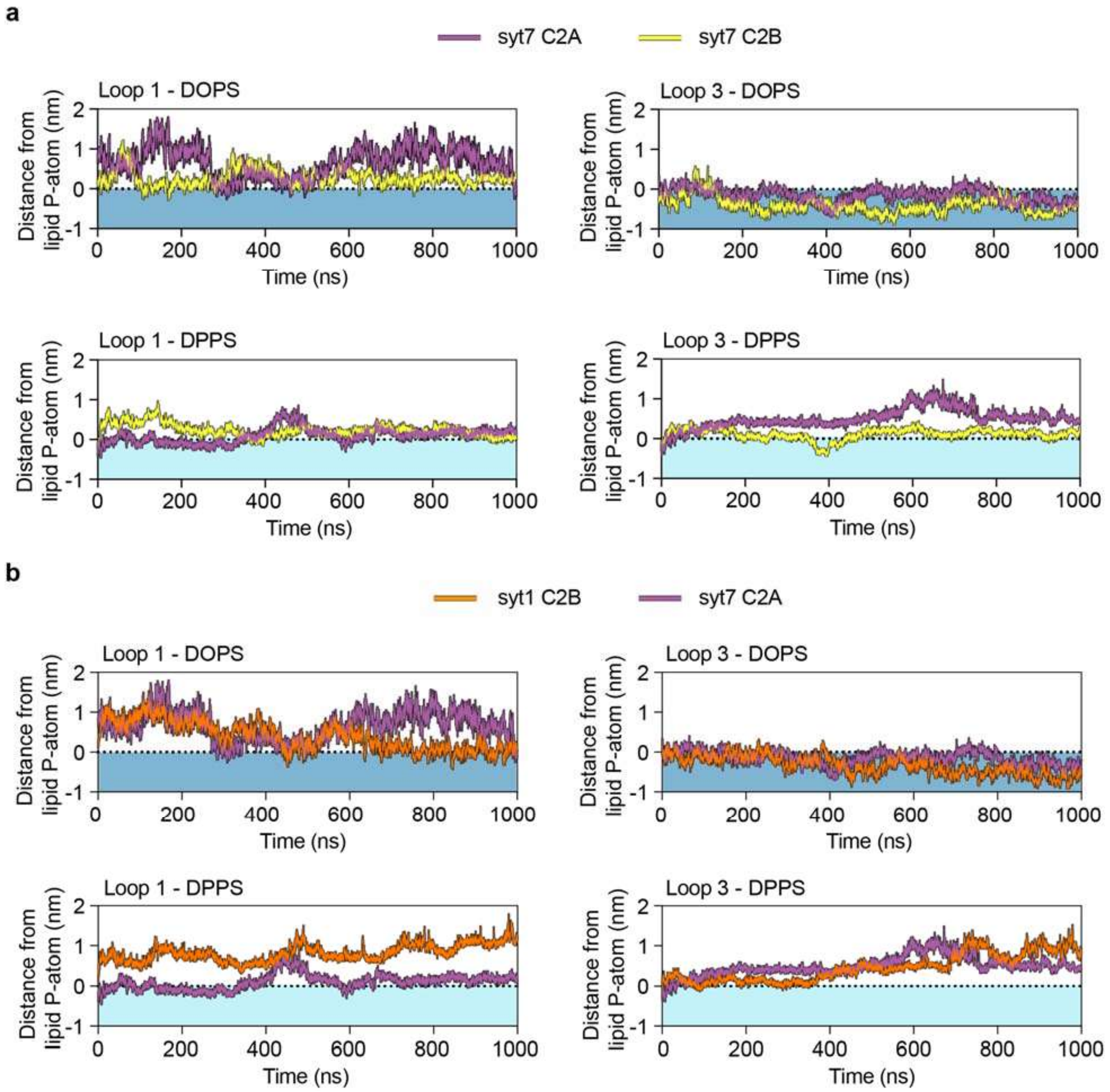

Revision Figure 3 (Revised manuscript Supplementary Fig. 7) – Pairwise MD simulations comparing the membrane penetration depth of syt7 C2A with syt1 and syt7 C2B domains in membranes containing DOPS or DPPS.

Next, we engineered and purified a new single cysteine syt7 C2AB variant to label loop 3 in the C2A domain and performed a new set of membrane penetration experiments, as described in Fig. 1. Specifically, we labeled syt7 C2AB at position 229 (F229C; C2A, loop 3), to complement the original labeling at position 361 in the C2B domain. The C2AB domain was then NBD labeled and membrane penetration was tested against bilayers composed of DOPC/DOPS or DOPC/DPPS (80:20). Like the C2B labeling results shown in Fig. 1, we found that the C2A domain of syt7 was also robustly able to penetrate membranes containing DPPS; the change in fluorescence that was observed after the addition of Ca^{2+} was nearly identical between DOPS and DPPS. These results, shown below and now included as Supplementary Fig. 2 in the revised manuscript, demonstrate that both syt7 C2-domains contribute to the

enhanced penetration activity of this isoform. These new penetration data, together with the new MD simulations dataset strongly support or original conclusion that syt7 is a super-penetrator C2-domain protein. We thank the reviewer for encouraging these endeavors as we feel these results have bolstered the rigor of our study.

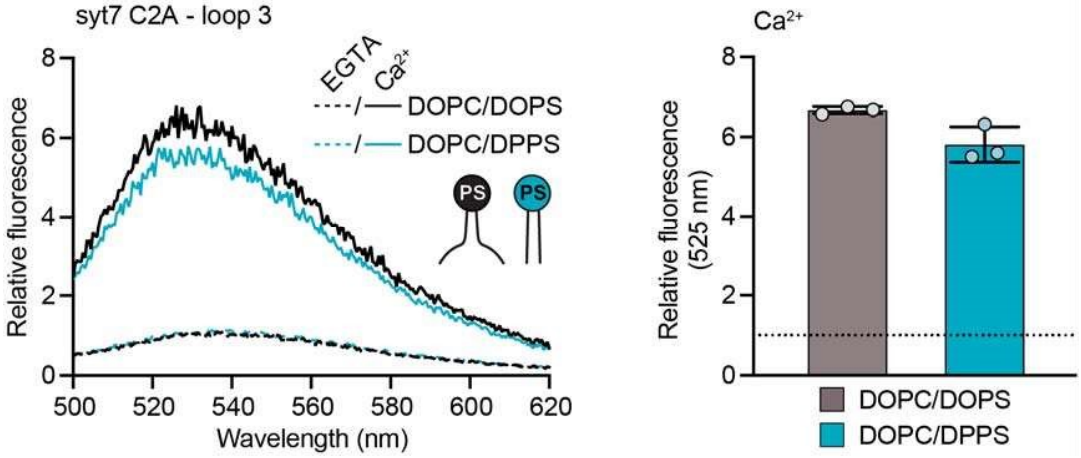

Revision Figure 4 (Revised manuscript Supplementary Fig. 2) – Membrane penetration by syt7 C2AB of bilayers composed of DOPC/DOPS or DOPC/DPPS (80:20). Loop 3 at position 229 in the C2A domain was labeled with NBD and penetration of 100 nm liposomes was compared in the absence (0.2 mM EGTA) and presence (0.5 mM free) Ca^{2+}.

2. How are saturated lipids distributed in cell membranes? Are their distributions correlated with the specific functions of syt1 and sy7 in synchronous and asynchronous exocytosis?

In the context of synaptic vesicle exocytosis, syt1 and syt7 target opposing membranes. Syt1 on the synaptic vesicle targets the plasma membrane, while syt7 on the presynaptic plasma membrane targets synaptic vesicles. Many synaptic vesicle components are derived from the plasma membrane via endocytic recycling, but these two surfaces are compositionally specialized.

In 2020, Lorent et al. published a clever study that examined the distribution of phospholipid acyl chains in the plasma membrane of erythrocytes (DOI: 10.1038/s41589-020-0529-6). This work concluded that the plasma membrane outer leaflet is highly enriched in saturated phospholipids. The inner leaflet, by contrast, contained primarily poly-unsaturated phospholipids. The most abundant phosphatidylserine was found to have 18:0-20:4 acyl chains. If this conclusion can be extrapolated to the neuronal plasma membrane, this suggests that the saturated phosphatidylserine used in our study (DPPS) is rare and thus unlikely to be directly relevant to syt1-membrane binding *in vivo*. However, these data did find a minor 16:0-18:0 phosphatidylserine component (less than 1%mol of the total) that, in principle, could influence exocytosis by assembling into clustered nanodomains at release sites.

Similarly, Takamori et al (2006) demonstrated that synaptic vesicle phosphatidylserine is highly poly-unsaturated (doi: 10.1016/j.cell.2006.10.030). The most abundant phosphatidylserine was reported to have a summed acyl chain structure of 40:6 (18:0-22:6 or 20:2-20:4, for example). This study implies that saturated phosphatidylserine is also unlikely to influence syt7 binding to synaptic vesicles *in vivo*.

In addition to the rarity of saturated phosphatidylserine, mammalian plasma membranes and synaptic vesicles contain approximately 30-40% cholesterol (https://pubmed.ncbi.nlm.nih.gov/2917977; doi:10.1111/j.1471-4159.1981.tb00435.x; doi: 10.1016/j.cell.2006.10.030). Our data demonstrated that cholesterol enabled syt1 to overcome

the defect in DPPS penetration. Therefore, unless release sites were both enriched in rare, saturated phosphatidylserine and are depleted of cholesterol, we expect syt1 penetration will be unencumbered by phosphatidylserine acyl chain structure *in vivo*.

It is important to note that while is it conceivable that presynaptic active zones have a specialized lipid composition that could include an enrichment of saturated phosphatidylserine, ultimately the phospholipid composition specifically within active zones is currently unknown. The possibility that phospholipid acyl chain structure locally influences exocytosis cannot be ruled out.

In our system, we viewed the use of DPPS as an experimental tool to better understand how C2-domains interact with phospholipid membranes; we did not intend to model a physiological protein-lipid interaction. Rather, by using phospholipid acyl chain structure to perturb syt-membrane interactions, this approach enabled us to perform experiments that examined how the act of membrane penetration by C2-domains contributes to their role in membrane fusion. This strategy specifically isolated the penetration component of the interaction, without relying on mutant proteins or the manipulation of other factors such as Ca^{2+} or SNARE binding that may have unintended consequences. We found that syt1, cPLA2, PKC and Doc2β all failed to penetrate membranes containing saturated phospholipids, whereas syt7 maintained robust penetration of DPPS membranes. The enhanced penetration function of syt7 then allowed us to correlate penetration performance with the ability of syt1 and syt7 to drive fusion pore dilation using nanodisc-black lipid membrane electrophysiology. These findings clearly establish the membrane penetration step as a crucial control point during fusion pore formation.

Finally, when considering this reviewer comment and the subsequent discussion that the most abundant phosphatidylserine species are poly-unsaturated, it was pertinent to validate in this response that syt1 can also penetrate poly-unsaturated phosphatidylserine membranes. All previous data compared C2-domiain interactions with either saturated or mono-unsaturated phosphatidylserine. Since poly-unsaturated phosphatidylserine is expected to be loosely packed, we anticipated efficient penetration, but this had yet to be tested in our system. For this, we revisited our C2-domain penetration assay using NBD-labeled syt1 and vesicles composed of DOPC and 18:2-18:2 phosphatidylserine. These experiments confirmed that syt1 is also capable of penetrating poly-unsaturated membranes. Although these data are not included in our revised manuscript, a representative result is shown below.

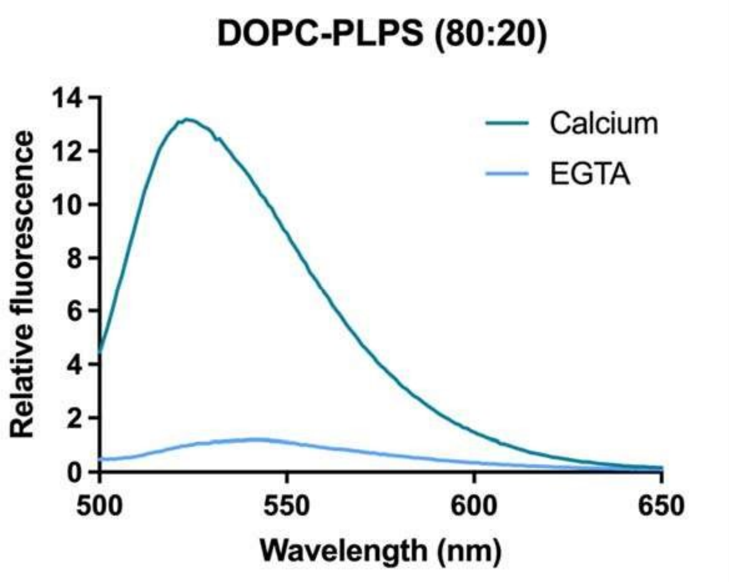

Revision Figure 5 – Representative trace showing syt1-NBD penetration into LUVs composed of DOPC (18:1/18:1) and polyunstaturated palmitoyl-linoleoyl-phosphatidylserine (16:0/18:2) in the presence of EGTA (0.2 mM) or 0.5 mM free Ca^{2+}.

3. In Fig. 2C, it will be helpful to highlight loop 1 and loop 3.

We appreciate the reviewer's suggestion and have modified Fig. 2 accordingly. A revised version of Fig. 2 is shown below. For consistency, we similarly modified Fig. 3a to include loop labels, also provided below.

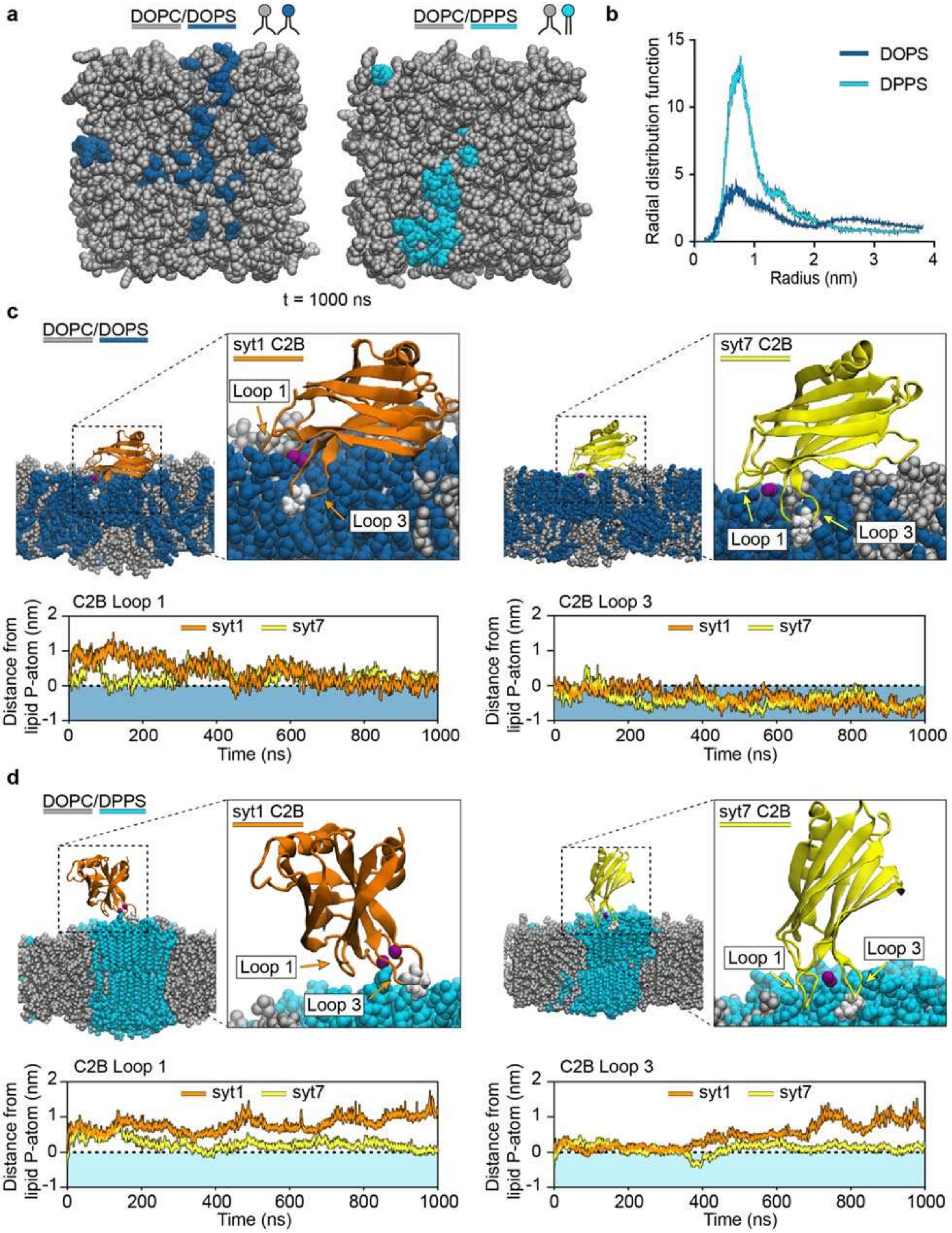

Revision Figure 6 (Revised manuscript Fig. 2)– To aid in the interpretation of Fig. 2, we included labels and arrows in panels C and D to clearly indicate the position of loops 1 and 3 in each representative MD simulation snapshot of the syt1 and syt7 C2B domains.

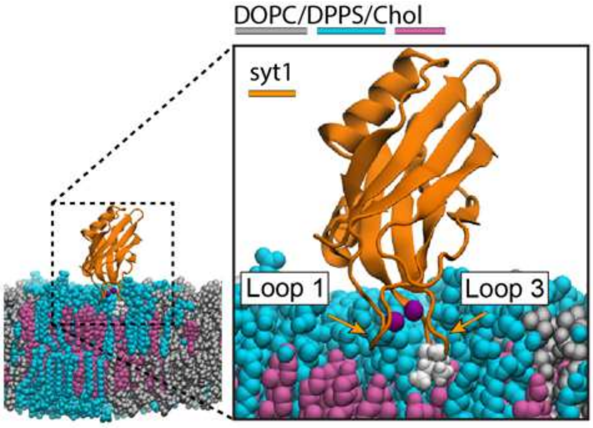

Revision Figure 7 (Revised manuscript Fig. 3a)– As with revised Fig. 2, Fig. 3a was modified to include labels and arrows to clearly indicate the positions of loop 1 and 3 of the syt1 C2B domain.

4. In Fig. 4A, it is not clear why LUVs precipitate. What buffer or reagents are put inside the LUVs that make them heavier in water?

The experiments summarized in Fig. 4 of our manuscript examined the binding of syt1 and syt7 C2AB domains, as well as various mutants, to 100 nm unilamellar vesicles. With the exception of Fig. 4d, and the right panels of Figs. 4h and 4i, the extent of membrane binding by the C2AB domains was determined via an ultracentrifugation-based co-sedimentation assay. The C2AB proteins are mixed with vesicles, with or without Ca^{2+} and subjected to centrifugation at 65,000 rpm for 60 minutes in a TLA-100 rotor. After centrifugation, the amount of protein remaining in the supernatant is visualized by SDS-PAGE and quantified by densitometry.

For these experiments, the high-speed centrifugation is sufficient to pellet the liposomes without the need to alter their weight, for example by sucrose encapsulation. The buffer inside and outside the vesicles are identical. To demonstrate the feasibility of this, we prepared a new sample of protein-free 100 nm unilamellar vesicles in 25 mM HEPES, 100 mM KCl (pH 7.4) that also contained 1% rhodamine labelled phosphatidylethanolamine for visualization. The image shows that after 60 minutes of centrifugation, the liposomes have been thoroughly pelleted.

We appreciate this reviewer comment as this highlighted that it would be more appropriate for our Methods section to report relative centrifugal force (x g), rather than RPM for this experiment. Due to the small diameter of the TLA-100 rotor, the high speed of 65,000 RPM equates to a robust 160,000 x g. The updated Methods section now reports 160,000 x g, in place of 65,000 RPM.

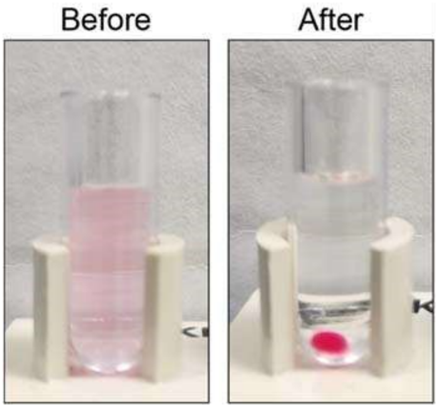

Revision Figure 8 – A demonstration that rhodamine labeled 100 nm unilamellar vesicles efficiently pellet after centrifugation for 60 minutes at 160,000 x g using a Beckman TLA-100 rotor.

5. In Fig. 5, it is not clear how the beautiful current trajectories are analyzed to derive the lifetimes of open and closed states and their probabilities. In principle, hidden Markov modeling can be used to unambiguously extract these properties (e.g., Zhang, Y.L., Jiao, J. & Rebane, A.A. Hidden Markov modeling with detailed balance and its application to single protein folding Biophys. J. 111, 2110-2124, 2016). These analyses will better support the PORE DILATION mediated by Syt1 and Syt7. In Fig. 5C, what's the current labeled on the y-axis? Is it the average current or the current of the open pore state? In Fig. 5D, the opening probabilities of Syt1 and Syt7 with DPPS are close to or slightly greater than 0.5, but the corresponding current traces in Fig. 5C show significantly higher opening probabilities. It will be better to show typical current traces.

First, we are grateful for these methodological questions; they revealed some references in the Figures and Methods documents that had been transposed. As such, the reference indicating how our planar lipid bilayer analyses were performed (doi: 10.1016/j.bpj.2021.02.005) could not readily be found. The references in the revised Figures and Methods documents have been corrected, and we apologize for this error.

For our planar lipid bilayer experiments, we record current passing through single fusion pores that form between a nanodisc that bears reconstituted v-SNAREs with planar lipid bilayers that bear reconstituted t-SNAREs. After approximately 30 minutes, the recording is stopped and the relevant data are extracted. The current values are determined by generating a current histogram for the open and closed states from each recording; we fit the histograms with a Gaussian function to identify the center of the peaks. The reported current values in Fig. 5c are the Gaussian fitted open state current values subtracted by the fitted near-zero closed state values.

For the kinetic analyses, the opening and closing transitions of the individual pores are analyzed using Clampfit software to determine the time that an opened pore remains in the open state and the time that an active fusion pore transiently reverts to the closed state. The results of these opening and closing transitions are then binned and plotted as open and closed dwell time histograms. From these data, we then also generate cumulative distribution functions for each individual recording and fit them with a single exponential using the following equation to derive the opening and closing transition rates: $CDF = 1 - \exp(-kt)$.

We agree with the reviewer that the approaches described in the provided Zhang, Jiao and Rebane reference could be appropriate and applied to our system. This powerful approach

has the ability to differentiate and quantify multiple transition states, which could reveal additional kinetic information in our experiments. Our ND-BLM recordings are typically characterized by single-step transitions between distinct open to closed states. We find Clampfit software to be adequate for analysis in this particular context. However, going forward, we are experimenting with pushing the limits of temporal resolution in our system. In these experiments, we expect additional intermediate states could be resolved and analyzed. We appreciate the reviewers for highlighting the hidden Markov modeling approach as Clampfit software may be inadequate for analyzing the data in these future experiments. When we obtain data at higher sampling rates, we may require alternative methods of analysis and the approach described in the Zhang, Jiao and Rebane study might be ideal; we will certainly investigate this.

Finally, regarding the comment relating the ND-BLM representative traces in Fig. 5b with the open probability values in Fig. 5d, we agree that the representative traces were not an ideal match for the open probability values. We revisited our data and selected new, more representative, raw traces for the syt7 and syt1 5W conditions. A revised Fig. 5 is shown below.

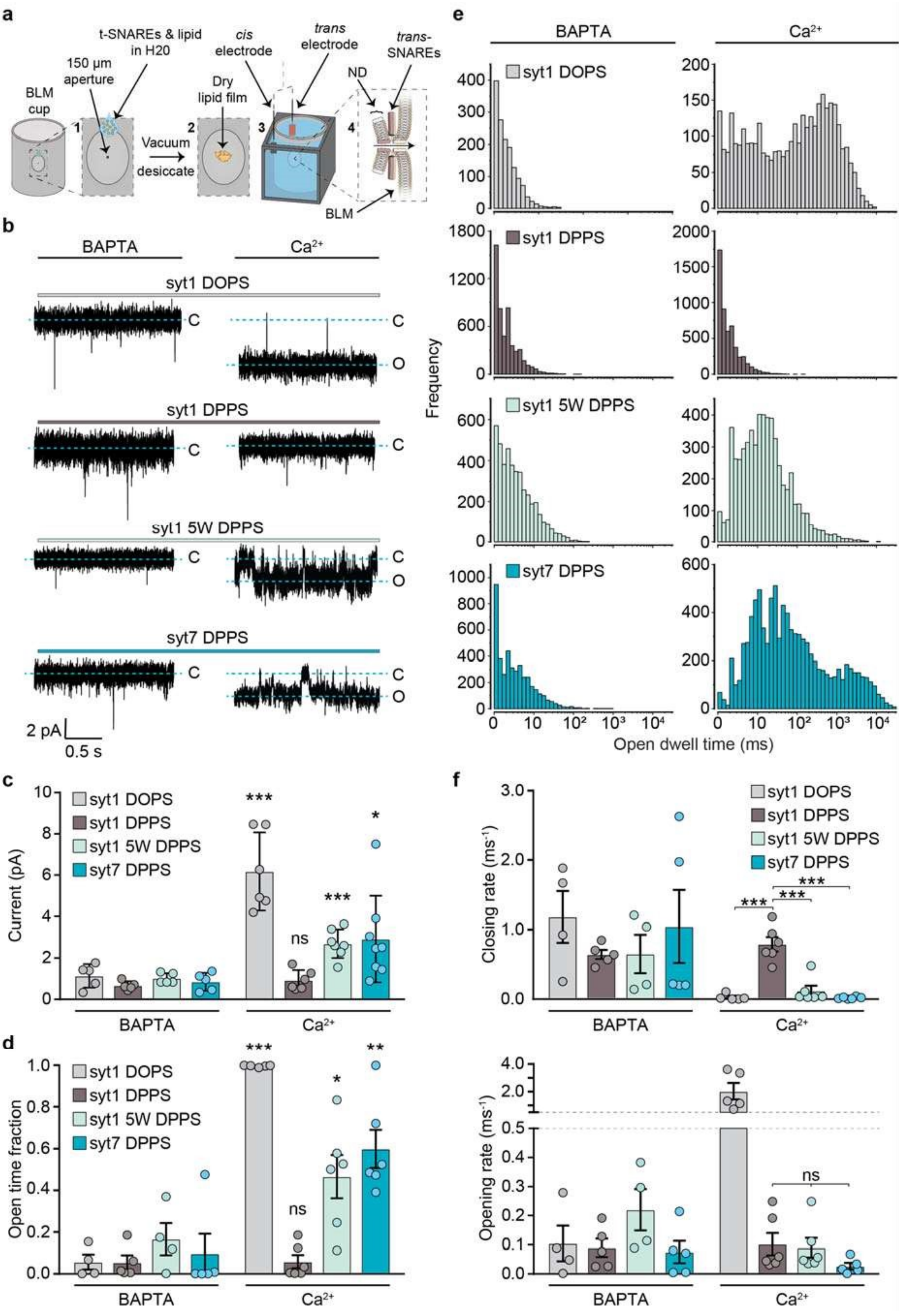

a
t-SNAREs & lipid in H20
150 µm aperture
BLM cup
Vacuum desiccate
Dry lipid film
cis electrode
trans electrode
ND
trans-SNAREs
BLM
b
BAPTA
Ca2+
syt1 DOPS
syt1 DPPS
syt1 5W DPPS
syt7 DPPS
C
O
2 pA
0.5 s
c
Current (pA)
ns
d
Open time fraction
e
Frequency
Open dwell time (ms)
f
Closing rate (ms-1)
Opening rate (ms-1)

Revision Figure 9 (Revised manuscript Fig. 5) – A reproduction of Fig. 5 showing new representative traces for syt1 5W and syt7 in panel b.

6. A reference may be needed for the sentence in lines 78-80.

We agree with the reviewer that a reference is warranted for the indicated sentence. We have updated the revised manuscript to now include the following reference:

Hui E, Gaffaney JD, Wang Z, Johnson CP, Evans CS, Chapman ER. Mechanism and function of synaptotagmin-mediated membrane apposition. *Nature Structural & Molecular Biology*. 2011/07/01 2011;18(7):813-821. doi:10.1038/nsmb.2075

Yongli Zhang and Jie Yang (Postdoc in the Zhang lab)

Reviewer #2 (Remarks to the Author):

Synaptotagmin 7 outperforms synaptotagmin 1 to promote the formation of large, stable fusion pores via robust membrane penetration

By Kevin C. Courtney, submitted to Nature Communications

The manuscript describes two important components in synaptic membrane fusion, the synaptotagmin 1 and 7. It illuminates how the distinct properties of the two proteins influence the pore formation related to membrane fusion. A combination of different techniques enables to identify the influence of membrane lipids thus entangling the influence of protein-protein interactions vs protein membrane interactions. A broad range of methods is used, fluorescence labelling to detect membrane penetration and membrane pore formation by a novel variant of black lipid membranes. The importance of synaptic vesicle fusion and the problems in understanding the mechanism makes the manuscript extremely relevant.
For other fields the combination of techniques and the novel information on this particular part of the SNARE system could extend our knowledge, further in membrane protein fields the approach is also highly innovative and interesting.
The manuscript is clearly written and the methods used are explained very well.
Some questions arise, however:

1. In the paragraph starting at line 201 the effect of cholesterol on syt1 penetration is described. At 30 % cholesterol complete membrane penetration is observed, however that seems like a very high cholesterol concentration under normal circumstances. Could the authors describe the physiological relevance of such high concentrations?

In Fig. 3 of our original manuscript, we demonstrated that the addition of cholesterol, both computationally and experimentally, enables syt1 to overcome the defect in DOPC/DPPS (80:20) membrane penetration. Fig. 3, panel e, reproduced below, shows that cholesterol can alter DPPS penetration by syt1 in a dose dependent manner, which increases to a plateau at 25% cholesterol (dashed orange line).

As with the phospholipid composition of the plasma membrane described above, the precise concentration of cholesterol specifically within exocytotic active zones is not known. However, the cholesterol content of mammalian cell plasma membranes (the target membrane of syt1) and synaptic vesicles has been previously characterized by multiple groups. Unanimously, the majority of the cellular cholesterol resides within the plasma membrane, but the exact %mol of cholesterol in this compartment varies in the literature between 25-40 %mol, depending on the experimental system (https://pubmed.ncbi.nlm.nih.gov/2917977; doi: 10.1016/j.ab.2009.07.027; doi: 10.1016/j.cell.2006.10.030). To avoid the margins of these estimates, we deemed 30% cholesterol a reasonable concentration as the maximum in Panel e.

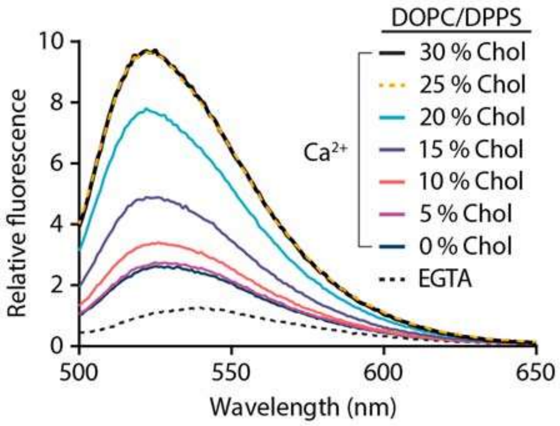

Revision Figure 10 (Revised manuscript Fig. 3e) – A reproduction of Fig. 3e showing how discrete increases in the concentration of cholesterol enables syt1 to overcome the defect in penetrating membranes contained 20% DPPS.

2. line 251: 5 W residues are substituted into the membrane penetration loops of syt1. It is argued that these residues make the loop hydrophobic and the observed effects are explained in terms of deeper membrane penetration because of increased hydrophobicity. There is quite a bit of literature that describes W as a lipid headgroup anchor, specifically localizing W in the headgroup regions of the lipid. Would that be a different explanation for the observed effects? Perhaps the manuscript should comment on this point or give arguments why the hydrophobicity is dominant in the case of the syt1 5W.

The syt1 5W data in our manuscript show that tryptophan substitutions in the penetration loops enable syt1 to overcome the defect in binding do DPPS membranes (Fig. 4e). We also demonstrated that syt1 5W is capable of binding membranes in the presence of elevated salt concentrations, unlike WT syt1 (Fig. 4g). We believe the enhanced features of the syt1 5W construct are the result of stronger interactions with phospholipids, compared to WT syt1. This enables full-length syt 5W to stabilize, and increase the size, of ND-BLM fusion pores formed in membranes containing DPPS (Fig. 5); again, a feat that WT syt1 cannot achieve.

In our original manuscript, we described the enhanced membrane binding by syt1 5W as increased hydrophobicity, however, when considering the reviewer's comment, we agree that tryptophan-phospholipid interactions can involve more than hydrophobic interactions. Indeed, there is considerable literature demonstrating that tryptophan residues can act as interfacial anchors for peptides and the transmembrane domains of proteins in lipid membranes. While strongly hydrophobic, tryptophan residues have an amphiphilic character that enable these residues to engage in multiple interactions with phospholipids (in addition to hydrophobic interactions), including interactions with the lipid phosphate and carbonyl groups. As a result of

this, tryptophan residues that are embedded in membranes are commonly enriched at the interfacial region.

Although tryptophan residues engage in interactions at the interfacial region of phospholipid bilayers, previous studies have demonstrated that tryptophan residues, engineered into the membrane penetration loops of C2-domains, will insert into the hydrophobic core of the bilayer. Specifically, when monitoring intrinsic tryptophan fluorescence, it has been demonstrated that the fluorescence of loop-localized tryptophan residues can be quenched upon binding of the C2-domains to liposomes containing membrane embedded doxyl-phosphatidylcholine (DOI: 10.1074/jbc.273.22.13995). Having a doxyl quencher at the depth of the 12th carbon position in the phospholipid acyl chains efficiently quenched the tryptophan fluorescence. This supports the conclusion that the tryptophan residues on C2-domain loops penetrate past the interfacial region of the bilayer (at least transiently). Another study found that soluble collision quencher, acrylamide, lost the ability to quench loop-labeled tryptophan fluorescence after C2-domain binding to liposomes (DOI 10.1074/jbc.M906729199*)*, again supporting that these tryptophan residues have penetrated past the surface of the bilayer to be shielded within the membrane.

Ultimately, the tryptophan substitutions in the syt1 5W construct likely promote strong membrane association by engaging in both hydrophobic and interfacial interactions with lipids. By substituting five tryptophan residues into the C2-domain penetration loops, multiple modes of phospholipid interactions would stabilize membrane binding and facilitate deeper penetration of the entire loop region. In summary, we agree with the reviewer that it would be appropriate in our manuscript to acknowledge the more complex membrane association behavior of these five tryptophan substitutions; we have modified the manuscript text accordingly. Specifically, when first introducing the syt1 5W construct, we now include both hydrophobic and interfacial interactions in the description, as follows:

"To delve into this further, we generated a series of mutants to examine how the hydrophobic and cationic character of the loops influenced binding. These included a syt1 C2AB construct that contained five tryptophan substitutions in the penetration loops (syt1 5W) to increase hydrophobic *and interfacial interactions with phospholipids*, or a poly-cationic mutant (L171K, H198K, K236R, N333R, Y364K, K369R) that substituted all the cationic residues of the syt7 loops on to syt1 (syt1 s7+)."

We appreciate the reviewer for raising this topic and for encouraging us to include a more accurate account of how the five tryptophan substitutions alter syt1-membrane interactions.

3. on line 312: 'nanodisc-black lipid membrane (ND-BLM)'. I find the term nano-disc in this context confusing: Usually nanodisk refers to nano-meter sized, solubilized planar membranes that are stabilized by a protein or polymer. In the context of the manuscript it seems that the expression nanodisc derives purely from the hole-size of the BLM cup. Could the authors explain? Or is that standard usage in the field of BLM membrane electric measurements? After taking into account the above minor points this manuscript is a very nice contribution, fit for publication in Nature Communications.

We apologize for the confusion on this topic and appreciate the opportunity to clarify here and in the revised manuscript. In short, we agree with the reviewer's description of the term 'nanodisc'. The nanodiscs in our case are 13 nm discoid lipid bilayers that are surrounded by a membrane scaffolding protein. We reconstituted v-SNARE proteins into these nanodiscs as a model membrane system to mimic v-SNARE-containing synaptic vesicles; t-SNARE proteins are similarly reconstituted into the black lipid membranes to mimic the plasma membrane. Upon

binding and zippering of v- and t-SNAREs together, *trans*-SNARE complexes are formed between the two membranes, leading to the opening of a fusion pore.

After revisiting the text of our original manuscript, we realize that some details of the nanodisc-black lipid membrane method were omitted. We instead provided citations to earlier work that thoroughly described the system. The only representation of a nanodisc in our original manuscript was an image of an ND-BLM fusion pore (Fig. 5a), which does not describe or depict events prior to the opening of the fusion pore. To clarify this issue, we included a new Supplementary Fig. 10 in our manuscript that describes two additional stages leading up to the opening of a fusion pore. This figure is reproduced below. We expect that inclusion of this new Supplementary Fig. 10 will resolve any future confusion regarding the definition of the term nanodisc in our experimental system. In addition, this new Supplemental Figure provides an opportunity to further describe how these experiments are performed. The revised manuscript has been expanded (new text indicated in italics) to state:

"Having established that PS acyl chain order can govern how C2-domains interact with lipid bilayers, we proceeded to investigate the functional consequences of restricting syt membrane penetration. We reasoned that PS acyl chain structure could be exploited to specifically interrogate the relationship between syt•membrane penetration and the ability of syts to promote membrane fusion[41]. For this, we used the recently developed nanodisc-black lipid membrane (ND-BLM) planar lipid bilayer electrophysiology approach[41, 55]. *This involves the reconstitution of v-SNARE proteins into a nanodisc (ND) to act as a SV mimic, while t-SNAREs are reconstituted into a planar lipid bilayer to mimic the presynaptic plasma membrane. When v-SNAREs in NDs associates with t-SNAREs in the supported bilayer, trans-SNARE complexes assemble to creates a nascent fusion pore across the two membranes (**Supplementary Fig. 10**). The properties of these fusion pores are then monitored electrophysiologically.* Previously, we reported that 13 nm NDs with three copies of the SV SNARE, synaptobrevin 2, (ND_3) make small unstable fusion pores[42, 55] with BLMs containing the t-SNAREs syntaxin-1A and SNAP-25B. However, incorporation of syt1 into these NDs caused the fusion pores to expand into larger, more stable structures in response to Ca^{2+} binding[41]. This experimental system is, therefore, ideal to examine the direct impact of syts on the properties of fusion pores. By substituting PS species within the BLM, we can examine how syt membrane penetration influences pore kinetic properties and dilation."

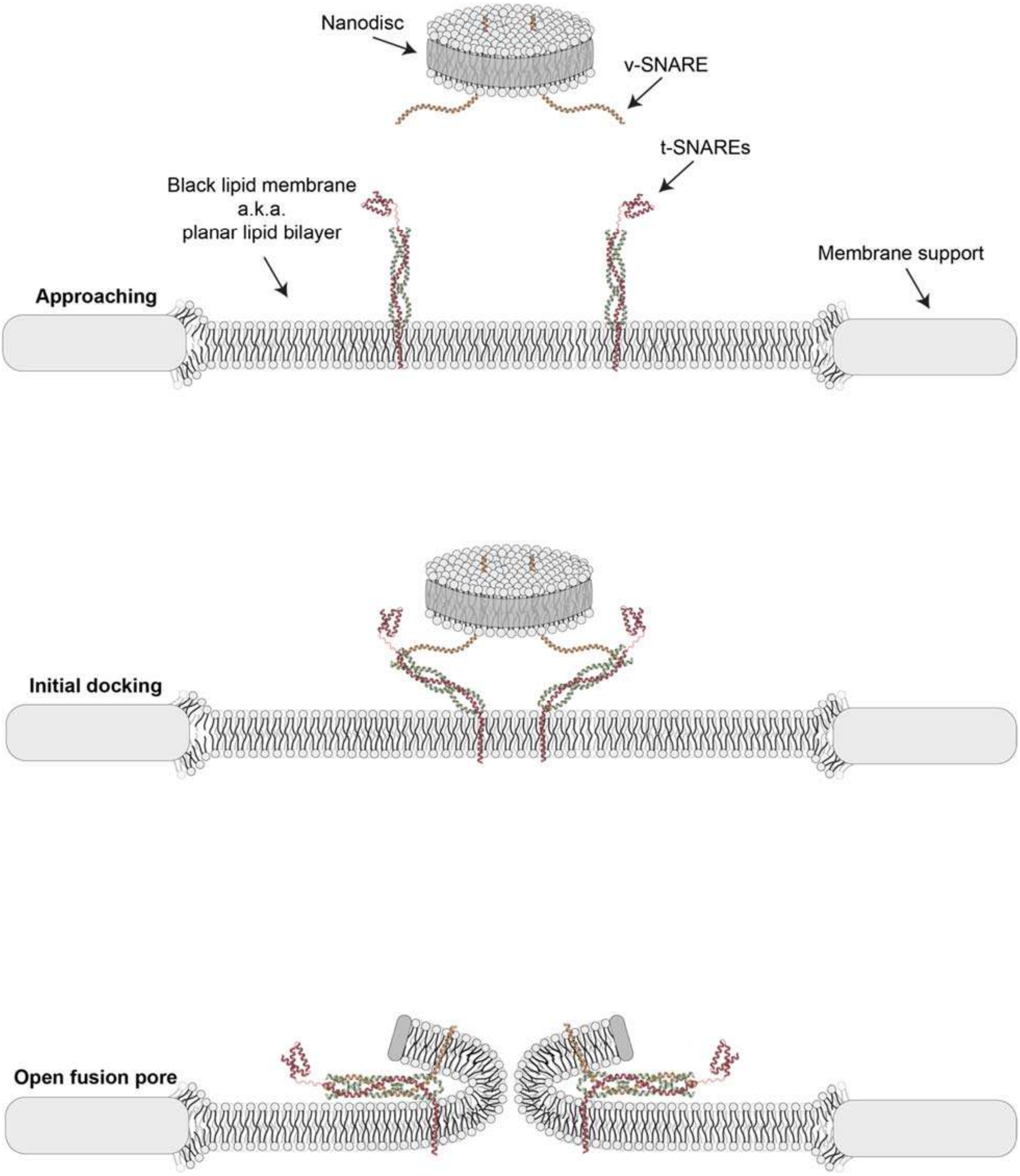

Revision Figure 11 (Revised manuscript Supplementary Fig. 10) – Depiction of the sequence of events leading up to the formation of a ND-BLM fusion pore. A v-SNARE nanodisc approaches, docks onto the t-SNARE BLM, and the SNAREs progressively assemble into *trans*-complexes that mediate the formation and opening of a fusion pore.

Reviewer #3 (Remarks to the Author):

In this report, the authors use biochemical and computational methods to demonstrate that the membrane interaction/penetration properties of the Synaptotagmin (Syt)-7 C2B domain is more robust than the Syt1 C2B domain. Using targeted mutations, they identify that the hydrophobic properties of the C2B acyl loops is a critical determinant of membrane penetrating activity. They further employ nanodisc-black lipid membrane (BLM) electrophysiology to correlate the membrane penetrating properties to the Syt1/7 ability to regulate fusion pores. This is a timely and well-written manuscript and provides crucial biochemical/molecular insights into the Syt7 protein. However, additional data is needed to understand the physiological/functional relevance of these findings.

1) Considering that the observed difference in membrane interaction activity of Syt1 and Syt7 is largely abrogated in the presence of physiological level (~30%) cholesterol in the lipid bilayer, it is unclear if the ascribed difference is physiologically and functionally relevant. It is likely that the Syt1 and Syt7 C2B domains might act very similarly under native cellular conditions especially since the authors do not provide any data on the behavior of Syt7 under cholesterol-containing conditions.

We agree with the reviewer that the presence of cholesterol in the target membrane would likely diminish the differences in membrane interaction activity observed by syt1 and syt7 in our experiments. As described in the response to comment #2 below, we believe that when membrane penetration is permissible in these reduced systems (i.e., not a limiting factor), the differential effects of syt1 and syt7 on their target membranes are more subtle. Please see below for direct comparisons from a newly added dataset. While our original datasets did reveal major functional differences in syt1 and syt7 activity when presented with the DPPS challenge, our study was multifaceted. In addition to differentiating syt1 and syt7 function, another primary objective of our study was to exploit the functional distinction between these two proteins for the purpose of assessing how the act of membrane penetration by C2-domains contributes to the syt-driven membrane fusion reaction. Identifying that syt1 and syt7 exhibited different penetration capabilities was crucial for this endeavor. We used lipid acyl chain structure as a tool to isolate the membrane penetration component from all other attributed roles of syt1, with syt7 and syt1 5W acting as gain-of-function comparators. In doing so, we found that membrane penetration is a critical component that enables syts to drive membrane fusion in response to binding Ca^{2+}.

Overall, however, our data demonstrate that syt7 exhibits enhanced membrane penetration, has higher affinity for binding phosphatidylserine (Supplementary Fig. 9) and is also far more resistant to elevated salt concentrations (Fig. 4g and Fig. 4i), compared to syt1. Although we do not expect that DPPS represents a true physiological challenge to syt7 *in vivo*, due to low abundance of this lipid species, we reiterate that the lipid composition at release sites is unknown. It is conceivable that release sites could be compositionally heterogeneous, favoring either syt1 or syt7 triggered fusion. This would be of particular importance regarding dense-core vesicle exocytosis since syt1 and syt7 have both been demonstrated to localize with dense-core vesicles in non-overlapping clusters (DOI: 10.1111/jnc.14986 and DOI: https://doi.org/10.7554/eLife.64527). Thus, the enhanced abilities of syt7 may enable this protein to drive dense-core vesicle fusion at release sites where syt1 falters.

In our study, we used DPPS and DOPS to address the question of whether syts must penetrate membranes, in response to Ca^{2+}, in order to regulate fusion pores. This is an important issue, as earlier work relied on mutations in syt, and these mutations also altered syt-SNARE interactions. Our new approach side-steps this weakness in the earlier work and demonstrates that the membrane penetration step is crucial for triggering the first step in fusion: the stable opening, and dilation, of a fusion pore. In the process of this work, we unexpectedly discovered that syt7 is a highly unusual C2-domain protein: it is a super-penetrator that can

insert into bilayers that are refractory to all other C2-domains tested. We feel this adds to the novelty of our study.

2) The authors have not included data on the effect of Syt7 on fusion pores formed with DOPS containing BLM. This is critical as the pore regulatory properties of Syt7 (e.g., current, open time fraction, opening rate shown in Figure 5C, D, F) under the DPPS condition are lower than that of Syt1 under DOPS conditions. Since Syt7 acts similarly under both DOPS and DPPS conditions, it stands to reason that Syt7 might actually be less efficient than Syt1 to stabilize the fusion pore under DOPS conditions.

The reviewer has made an interesting observation here that warranted further study. Specifically, our nanodisc-black lipid membrane electrophysiology data showed that syt1 fusion pores, formed in bilayers containing DOPS, are larger and more stable than pores formed by syt7 in bilayers containing DPPS. As stated by the reviewer, since syt7 penetration is comparable between DOPS and DPPS, it is a reasonable hypothesis that syt7 could be less efficient at opening and stabilizing pores in DOPS membranes, compared to syt1. The original version of our manuscript did not include a direct comparison of syt1 and syt7 fusion pores in membranes containing DOPS; we now address this issue and report these results in a new Supplementary Fig. 12, reproduced below.

The single-pore experimental results outlined in Fig. 5 of our manuscript are exceptionally slow to acquire a complete dataset and are often impractical to produce thoroughly during revision. Fortunately, we had already been in the process of generating a new set of planar lipid bilayer electrophysiology experiments examining the effect of syt1 and syt7 on the properties of fusion pores formed in bilayers containing DOPS. These experiments were performed using conventional methods of forming planar lipid bilayers (i.e., painting lipids in n-decane), rather than the novel drying method described in Fig, 5a that was developed to enable studies with DPPS.

As expected with the membranes containing DOPS, we found that the addition of Ca^{2+} caused both syt1 and syt7 to increase fusion pore size and to stabilize the open state of the pores, as compared to the Ca^{2+}-free condition. The Ca^{2+}-free conditions were statistically equal between syt1 and syt7. Interestingly, comparative analysis of syt1 and syt7 in Ca^{2+} was also largely the same throughout; the only statistically significant difference in the entire dataset was found between the current values in Ca^{2+}. Specifically, Ca^{2+}-syt7 yielded significantly higher currents than Ca^{2+}-syt1. We note that two trials in the Ca^{2+}- syt1 condition resulted in rather small and unstable pores. These trials are quite apparent when looking at the open time fraction and kinetic analyses plots. These traces had the effect of left-shifting the open dwell time distribution in this condition (brown bars, panel F) and somewhat dwarfing the longer open times. While these points are outliers in most of the analyses, we elected to include all the acquired data in the Figure. Regardless, the open dwell time histograms of both syt1 and syt7 clearly show the emergence of significantly longer open times in response to Ca^{2+}. The kinetic analyses, derived from the open and closed dwell time distributions found syt1 and syt7 were comparable.

Taken together, the results of our new nanodisc-black lipid membrane electrophysiology experiments show that syt7 is not less efficient than syt1 when acting upon bilayers containing DOPS, as hypothesized based on our original dataset. When interacting with membranes, where bilayer penetration is not limiting, syt1 and syt7 exert comparable effects on the properties of fusion pores, with syt7 generating slightly larger pores. The kinetic differences between syt1 and syt7 were only revealed when penetration was hindered by the use of DPPS, thus becoming a limiting factor in the reaction.

Knowing now that syt7 is not less efficient that syt1 in DOPS, we speculate that the differences between syt1 in DOPS and syt7 in DPPS, observed in the original version of our

manuscript, are likely due to other effects of DPPS on the biophysics of the bilayer; syt penetration is only one aspect of the Ca^{2+}-triggered fusion reaction. Our AFM data show that Ca^{2+} causes DPPS to assemble into tight clusters (Supplementary Fig. 5). In this case, the syts would be binding clustered phosphatidylserine. Thus, we believe that the fusion pore site(s) in the DPPS nanodisc-black lipid membrane experiments are likely enriched in phospholipids with saturated acyl chains. Fusion pores are characteristically highly curved structures, and we believe that pores formed with rigid, saturated acyl chains would be inherently less efficient than pores formed by membranes with flexible, unsaturated chains (DOPS). While syt7, and syt1 5W, exhibited similar penetration performance into DOPS and DPPS containing membranes, the presence of saturated DPPS acyl chains in the fusion pore likely increases membrane rigidity, which would restrict pore dilation; this increase in membrane rigidity would likely contribute to the observed differences between syt1 in DOPS and syt7 in DPPS. Overall, however, our data demonstrate that when syts are capable of penetrating the target membrane in response to Ca^{2+} -- syt1 in DOPS, syt1 5W or syt7 in DPPS -- syts can stabilize the open state, and drive dilation of, fusion pores.

The results section of our revised manuscript now includes the following text to accompany the new Supplementary Fig. 12:

"The ND-BLM data presented thus far exclusively employed our newly developed method of forming planar lipid bilayers via drying and re-hydrating the lipids across the aperture of the BLM cup (**Fig. 5A**); this facilitated the incorporation of DPPS into the BLM and enabled us to study how syt penetration impacts fusion pore properties (**Fig. 5**). Next, we reverted to classical BLM painting methodology[41, 55] to make a direct comparison of syt1 and syt7 when both proteins were capable of penetrating the target membrane in response to binding Ca^{2+}. These experiments used ND_3-syt1 and ND_3-syt7 in association with DOPS-containing t-SNARE BLMs. Using this approach, syt7 continued to generate significantly larger fusion pores than syt1, but the kinetic analysis found no significant differences in open time fractions, or the closing and opening rates of the pores that are regulated by these two proteins (**Supplementary Fig. 12**). Thus, when membrane penetration is no longer a limiting factor, the functional differences in syt1 and syt7 are more subtle under these conditions."

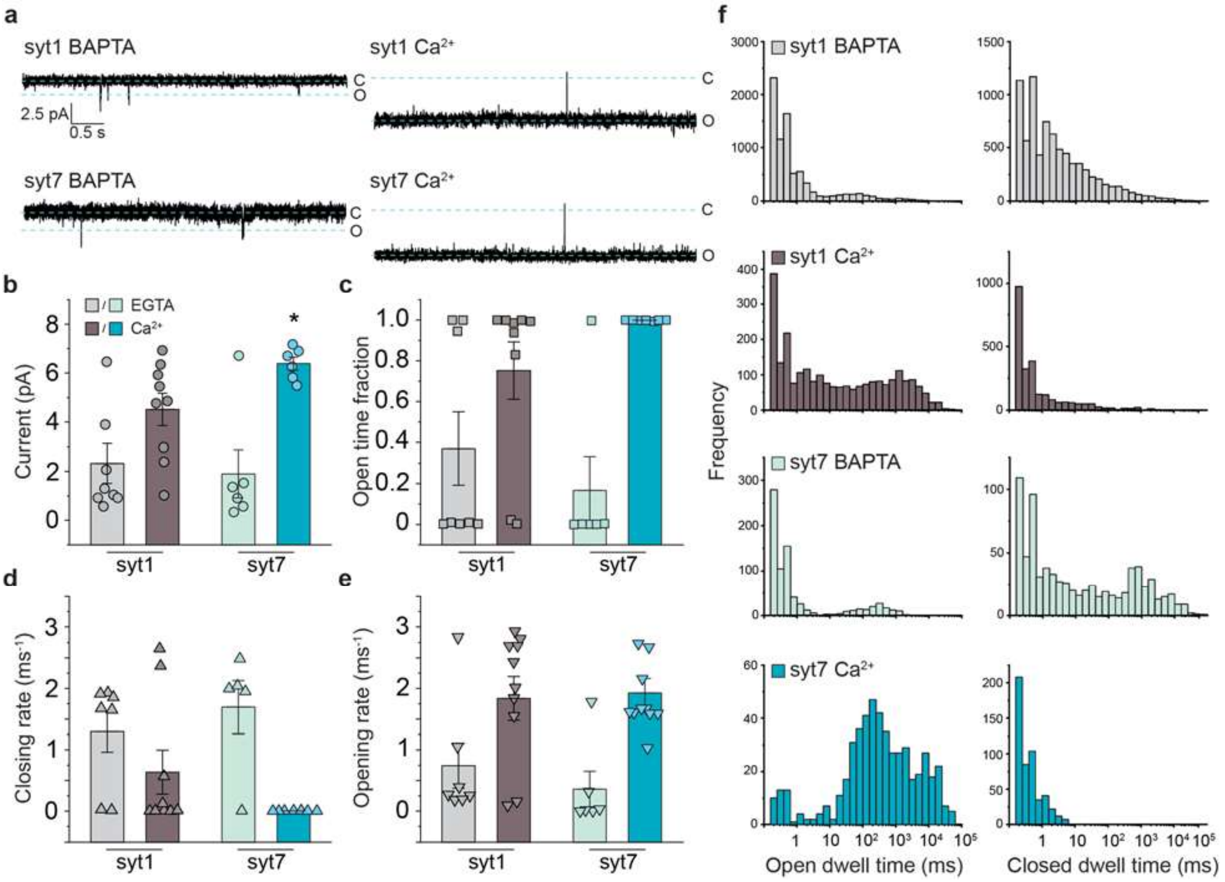

Revision Figure 12 (Revised manuscript Supplementary Fig. 12) – A new dataset showing how syt1 and syt7 affect ND-BLM fusion pore properties in membranes containing unsaturated DOPS.

3) The manuscript is focused on the C2B domain of the Syt isoforms. While the C2B domain is critical for the Syt1 function, it is well-established that the Syt7 C2A domain is crucial to its function. Thus, it might be important to understand how the C2A domain of Syt7 compares to the Syt1 C2B domain.

We appreciate the reviewer's suggestion to compare the membrane interactions of the C2A domain of syt7 with the C2B domain of syt1. This is an important point that was shared by Reviewer #1, addressed in detail above. The original version of our manuscript compared the C2B domains of syt1 and syt7 in Figs. 1 and 2, with the remainder of the manuscript examining the C2AB domains or full-length proteins. As mentioned above, we initially thought it was logical to compare the membrane interactions of syt1 and syt7 by monitoring penetration at the same relative positions in the two proteins. Having done this, we agree with the reviewer that the manuscript would greatly benefit from a more detailed examination of the syt7 C2A domain. As described above with reviewer #1, comment 1, we generated new MD simulations with the syt7 C2A domain interacting with DOPS or DPPS membranes and a new experimental dataset examining membrane penetration by C2A-labeled syt7 C2AB. A thorough description of these data are above, and we have reproduced this response below for convenience. We appreciate the reviewer's suggestion to include these additional data; our manuscript now includes a more comprehensive and detailed examination of the syt7 C2A and C2B domains.

Reproduction of the response to reviewer #1, comment 1:

1. The authors appear to focus on the C2B domain in both Syt1 and Syt7. However, it is reported that Syt7 C2A domain dominates the membrane binding (Voleti et al., PNAS 2017). What is the role of C2A in membrane penetration in a saturated lipid bilayer?

This is an important comment that was shared by reviewers 1 and 3. The reviewers are correct to point out that aspects of our original manuscript compared membrane interactions of the syt1 and syt7 C2B domains. Specifically, the membrane penetration experiments in Fig. 1 placed the NBD reporter on the third loop of the C2B portion of each C2AB domain and the MD simulations shown in Fig. 2 only modeled the C2B domains. The remainder of the comparisons of syt1 and syt7 in our study assayed the C2AB domains or full-length proteins, largely indiscriminate of the individual C2-domains. The sole exception to this was in Fig. 4g and Fig. 4i, where we tested syt7 C2AB mutants with reduced loop hydrophobicity in either the C2A or C2B domains (syt7 $C2_{2A}B$ and syt7 $C2AB_{2A}$). These experiments support the importance of the C2A domain as we found that reduced hydrophobicity in syt7 C2A had significant impact on membrane binding. Reducing loop hydrophobicity in the C2B domain however, had no discernable effect. A reproduction of these data panels is shown here:

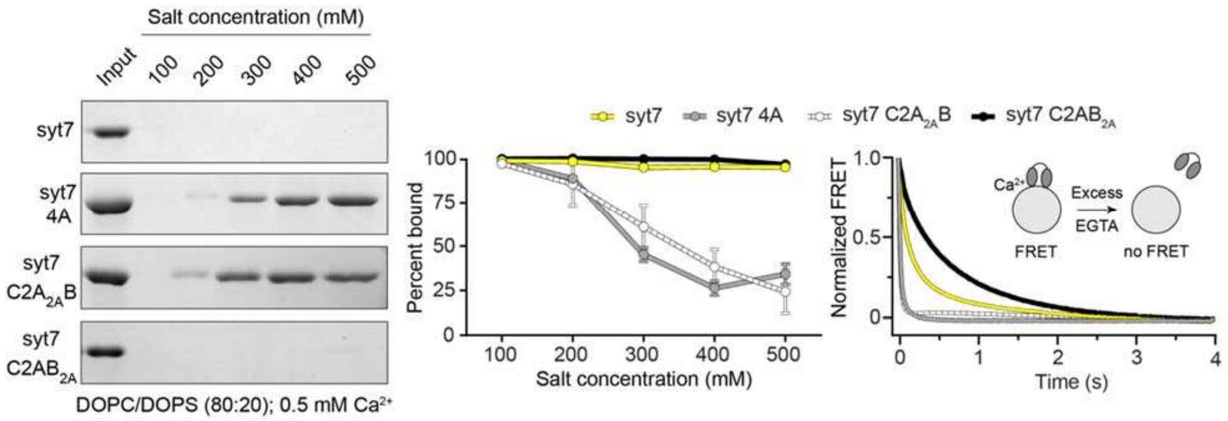

Revision Figure 1 – Reproduction of data panels from Fig. 4.

In designing this study, we found it logical to begin comparing syt1 and syt7 by monitoring their interactions with membranes at the same relative positions, in their 3-dimensional structures, for Figs. 1 and 2 (C2B, loop 3). However, the reviewers raise an interesting question that was not specifically addressed in our original manuscript. Namely, how does the C2A domain of syt7 perform against a DPPS-containing membrane? To directly address this issue, we generated new computational and experimental datasets to assess syt7 C2A-membrane interactions as a complement our original datasets.

Computationally, we generated a new set of MD simulations of the syt7 C2A domain interacting with membranes composed of DOPC/DOPS (80:20) or DOPC/DPPS (80:20); these simulations were carried out using the same parameters as our original computational efforts in Fig. 2. The C2A domain was placed at the membrane surface and allowed to equilibrate for 1000 ns. As expected, we found that the syt7 C2A domain was able to efficiently penetrate the bilayer containing DOPS. Interestingly, loop 3 of the syt7 C2A domain dominated the DOPS-membrane interaction, causing the C2A domain to tilt, restricting the depth of loop 1 penetration. When examining the interaction of syt7 C2A with the DPPS bilayer, our analysis again showed membrane penetration, which is in sharp contrast to the syt1 C2B domain. Loop 1 of the syt7 C2A domain penetrated well to the depth of lipid phosphate groups, while loop 3 penetration

was on average, 0.5 nm above the phosphate layer engaging in interactions with the lipid head groups. An illustration of these new MD simulations is shown below (and these finding are also shown in Supplementary Fig. 6 of our revised manuscript).

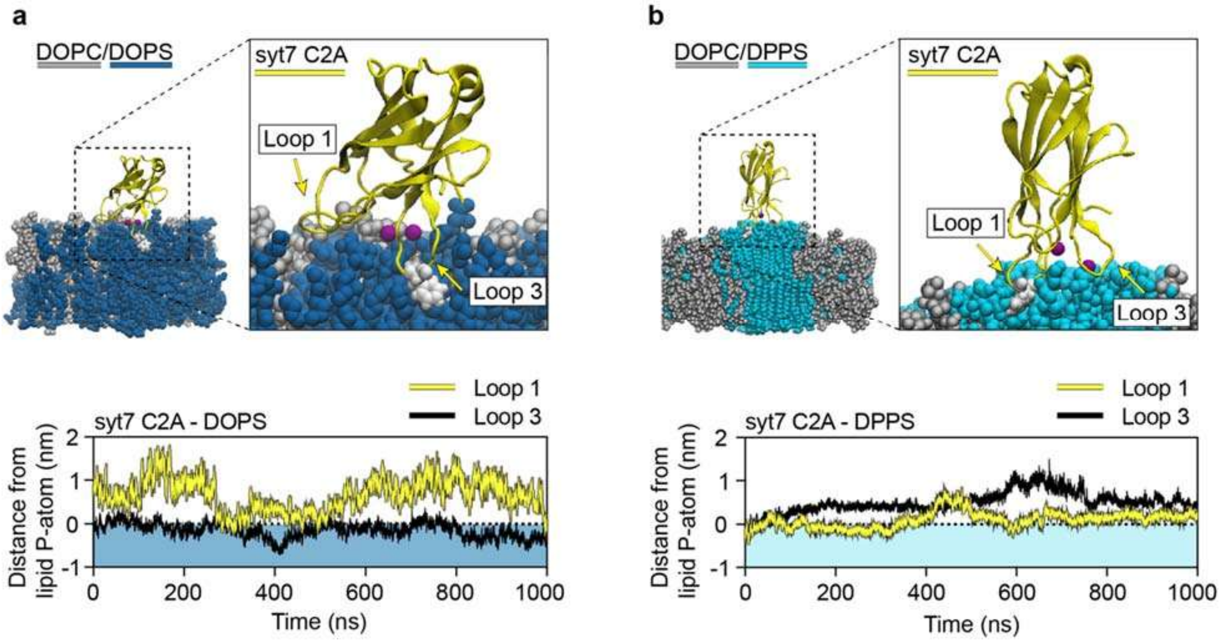

Revision Figure 2 (Revised manuscript Supplementary Fig. 6) – MD simulations of the syt7 C2A domain interacting with lipid bilayers containing DOPS or DPPS. A new set of MD simulations with syt7 C2A were performed to complement our original syt1 and syt7 C2B datasets.

With this new set of syt7 C2A MD simulations, we were then able to make additional pairwise comparisons. We generated a new supplementary figure (now Supplementary Fig. 7), shown below, that compares loop 1 and 3 penetration depth between syt7 C2A and C2B domains, as well as the syt7 C2A domain with the C2B domain of syt1. These additional pairwise comparisons show that each C2-domain that we tested efficiently penetrates DOPS membranes; only loop 1 of syt7 C2A largely remains on the DOPS membrane surface, due to tilting of the C2-domain.

In DPPS, both syt7 C2-domains exhibited similar membrane penetration. The penetration depth of loop 1 was nearly identical for both syt7 C2-domains, while loop 3 of C2A was slightly less than syt7 C2B. When comparing syt1 C2B with syt7 C2A in DPPS, a clear distinction is observed for loop 1; syt7 C2A is superior as syt1 C2B fails to penetrate. In contrast, loop 3 of syt1 C2B and syt7 C2A are more similar. Together, these additional MD simulations demonstrate that both C2-domains of syt7 contribute to efficient penetration into DPPS membranes.

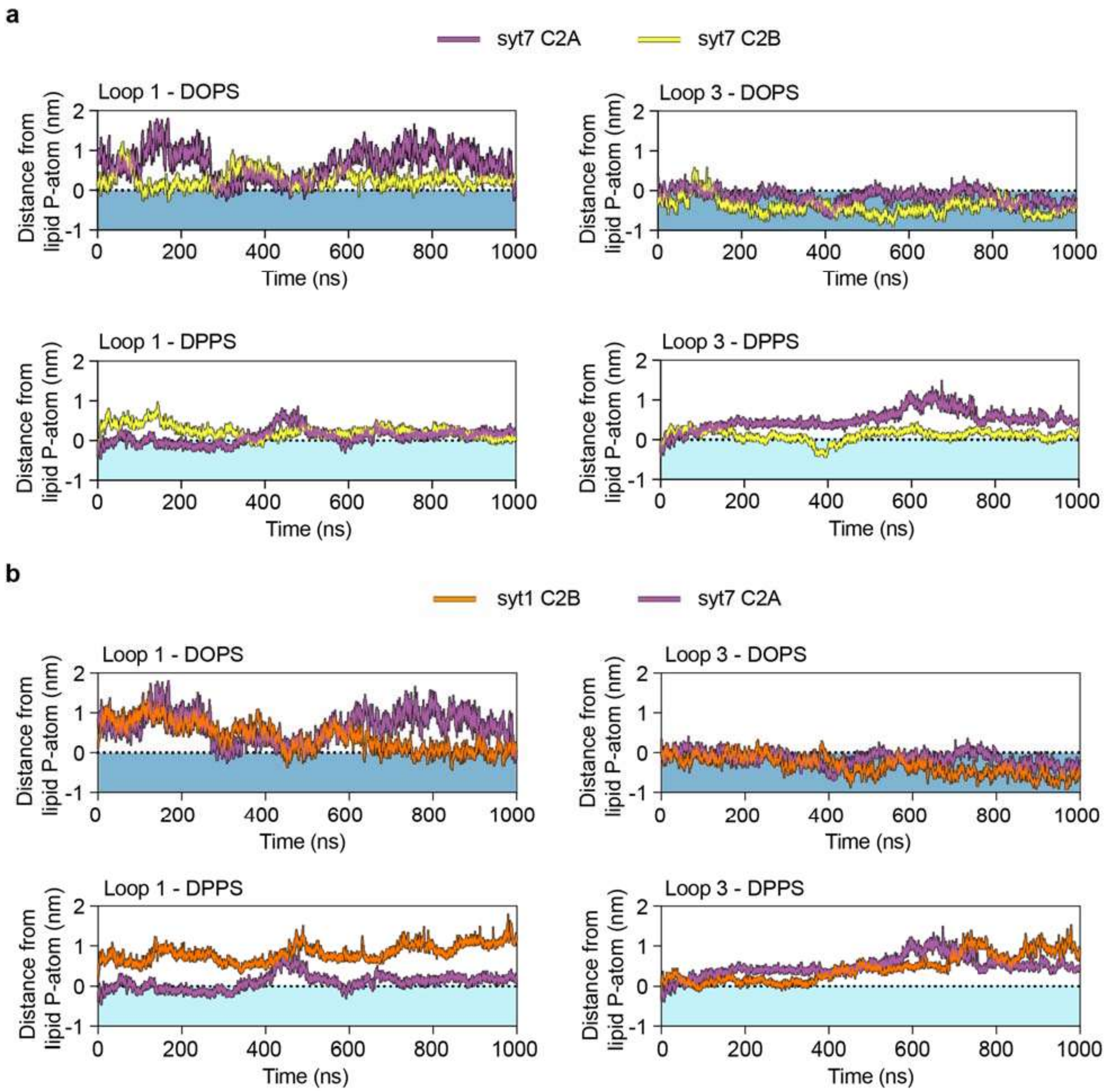

Revision Figure 3 (Revised manuscript Supplementary Fig. 7) – Pairwise MD simulations comparing the membrane penetration depth of syt7 C2A with syt1 and syt7 C2B domains in membranes containing DOPS or DPPS.

Next, we engineered and purified a new single cysteine syt7 C2AB variant to label loop 3 in the C2A domain and performed a new set of membrane penetration experiments, as described in Fig. 1. Specifically, we labeled syt7 C2AB at position 229 (F229C; C2A, loop 3), to complement the original labeling at position 361 in the C2B domain. The C2AB domain was then NBD labeled and membrane penetration was tested against bilayers composed of DOPC/DOPS or DOPC/DPPS (80:20). Like the C2B labeling results shown in Fig. 1, we found that the C2A domain of syt7 was also robustly able to penetrate membranes containing DPPS; the change in fluorescence that was observed after the addition of Ca^{2+} was nearly identical between DOPS and DPPS. These results, shown below and now included as Supplementary Fig. 2 in the revised manuscript, demonstrate that both syt7 C2-domains contribute to the

enhanced penetration activity of this isoform. These new penetration data, together with the new MD simulations dataset strongly support or original conclusion that syt7 is a super-penetrator C2-domain protein. We thank the reviewer for encouraging these endeavors as we feel these results have bolstered the rigor of our study.

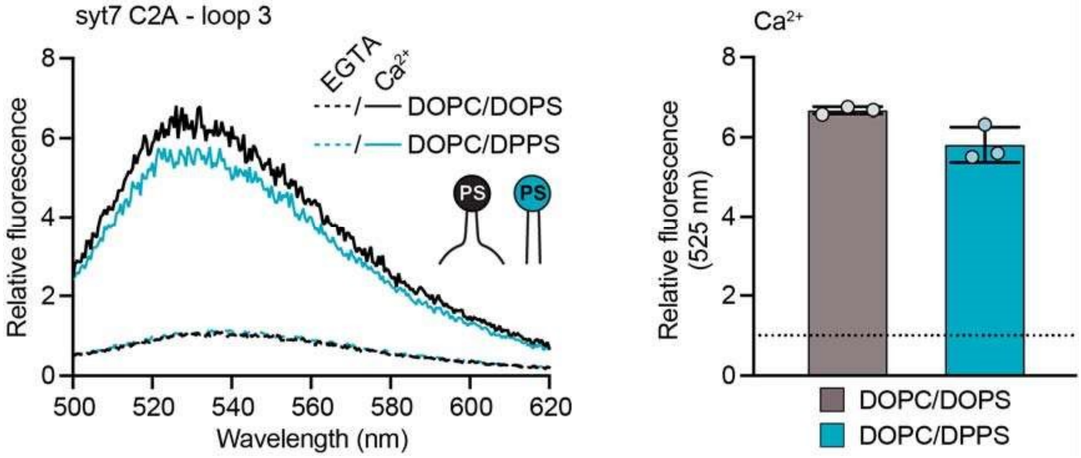

Revision Figure 4 (Revised manuscript Supplementary Fig. 2) – Membrane penetration by syt7 C2AB of bilayers composed of DOPC/DOPS or DOPC/DPPS (80:20). Loop 3 at position 229 in the C2A domain was labeled with NBD and penetration of 100 nm liposomes was compared in the absence (0.2 mM EGTA) and presence (0.5 mM free) Ca^{2+}.

REVIEWERS' COMMENTS

Reviewer #1 (Remarks to the Author):

The authors have performed more experiments, carefully revised the manuscript, and properly addressed the concerns raised by reviewers. This is a very beautiful piece of work. I support its publication in NC without further changes.

Reviewer #3 (Remarks to the Author):

The authors have addressed my/other reviewers' queries and I recommend the publication of the revised manuscript.
